# Minimum chi-square method for estimating population size in capture-recapture experiments

**DOI:** 10.1371/journal.pone.0292622

**Published:** 2023-10-12

**Authors:** Yuyan Zheng, Yongfei Mao, Min Tsao, Laura L. E. Cowen

**Affiliations:** Mathematics and Statistics, University of Victoria, Victoria, British Columbia, Canada; Utrecht University: Universiteit Utrecht, NETHERLANDS

## Abstract

Closed population capture-recapture estimation of population size is difficult under heterogeneous capture probabilities. We introduce the minimum chi-square method which can handle multi-occasion capture-recapture data. It complements likelihood methods with elements that can lead to confidence intervals and assessment of goodness-of-fit. We conduct a comprehensive study on the minimum chi-square method for estimating the size of a closed population using multiple-occasion capture-recapture data under heterogeneous capture probability. We also develop two different bootstrap techniques that can be combined with any underlying estimator, be it the minimum chi-square estimator or a likelihood estimator, to perform useful inference for estimating population size. We present a simulation study on the minimum chi-square method and apply it to analyze white stork multiple capture-recapture data. Under certain conditions, the chi-square method outperforms the likelihood based methods.

## Introduction

Capture-recapture methods are commonly used in wildlife studies to estimate population size by using information from marked individuals. The Lincoln-Petersen method [1] is the simplest method for estimating a population size with capture-recapture data. Many authors have proposed extensions to model capture-recapture data from a closed population (where the population is closed to births/deaths and immigration/emigration) [2], including developments to incorporate capture probabilities that vary over individual (heterogeneous capture probabilities). For example, [3] suggested using the full and conditional likelihood methods to estimate population size; [4] recommended using a jackknife estimator; [5] proposed a conditional likelihood estimator with covariates; [6] suggested likelihood estimation with finite mixture models; and [7] investigated a Conway-Maxwell-Poisson estimator as it allows different levels of heterogeneity adaptively.

Link [8] described the nonidentifiability of a population size *N* as the case where populations of very different sizes give rise to roughly the same observed capture history, which implies when we have only the capture history as we normally do in a capture-recapture experiment, the underlying population size cannot be accurately determined. Link (2003) brought forth the nonidentifiability problem for population size *N* from the standpoint that *N* could not be identified based on the conditional distribution of the observed data. He tried maximizing the full likelihood function and maximizing the conditional likelihood function to estimate population size. In essence, the nonidentifiability problem refers to the over-dependence; in the sense that for a given set of data, the estimated value $\hat{N}$ can be vastly different when the underlying capture probability distribution g(*p*; ***θ***) changes. In recent wildlife studies, researchers have focused on the distribution of capture probability *p* to model the heterogeneities and estimate the abundance under heterogeneity. The authors of [9] utilized a logit-normal model for *p* and [10] recommended using a beta model for *p*. The problem of nonidentifiability under heterogeneity still remains when setting high detection probabilities, restricting the number of parameters in models, or obtaining a large number of sampling occasions [11].

We explore the minimum chi-square method and compare it to two existing likelihood methods for estimating population size using multi-occasion capture-recapture data, and we apply these to estimate the white stork (*Ciconia ciconia*) population size in their main European wintering area, southwestern Spain. A white stork data set was obtained from [12], who focused on analyzing individual consistency in the use of food subsidies. Individuals within the study population were detected daily throughout the wintering season lasting 80 days with the additional state information referring to the foraging categories (dumps or rice fields) in which the individuals were detected. The authors of [13] commented that there were about 4000 white storks wintering in the study area when the capture history data were recorded. This number is based on census data collected and synthesized by scientists from the Spanish Ornithological Society in collaboration with Birdlife International. We give complementary estimates based on the three statistical methods of estimation. The existing likelihood methods that we use are the full and conditional likelihood methods. The minimum chi-square method is a recognized alternative to likelihood based methods [14]. It has been applied to capture-recapture data on a small humpback whale study assuming constant capture probability [15] and was investigated by [16] in the context of time varying capture probabilities. Authors of [17] mentions the possibility of using the minimum chi-square method in a three sample closed population study, but never applied it to their study. We are interested in this method because it lends itself naturally to the multi-occasion capture-recapture data which are categorical data that can be directly handled by this method without discretization. Further, a major advantage to the minimum chi-square method is that it provides point estimation, interval estimation and goodness-of-fit assessment at the same time. We adopt this method for multi-occasion capture-recapture data and show through the white stork data analysis that it is a competitive alternative to existing methods.

The rest of this paper is organized as follows. In Section 2, we first give a brief review of the full and conditional likelihood methods for multi-occasion capture-recapture data, and then discuss and adopt the minimum chi-square method for such data. We also propose two bootstrap techniques for multi-occasion capture-recapture data which can be used to construct confidence intervals for the population size. In Section 3, we present a simulation study to examine the finite sample properties of the minimum chi-square statistic which depend on the underlying population size and sample size. Due to our interest in estimating the white stork population size, we will focus on the case where the population size is not too large. We then apply the likelihood and the minimum chi-square methods to estimate the white stork population size in Section 4 and conclude with some remarks in Section 5. We also studied the accuracy and robustness of the minimum chi-square method when the underlying population is much larger than the white stork population. Results concerning such large population cases are given in the S1 File.

## Materials and methods

### The full and conditional likelihood methods

We review the full and conditional likelihood methods by following the discussion about these in [11]. Let *N* be the unknown population size, *X*_1_, *X*_2_, …, *X*_*N*_ be independent random variables where *X*_*i*_ is the number of times individual *i* is observed, and *T* be the number of sampling occasions. Define a vector of frequencies ***f*** = (*f*_0_, *f*_1_, …, *f*_*T*_)′ where *f*_*x*_ = #(*X*_*i*_ = *x*) for *x* = 0, 1, 2, …, *T* and let *p*_1_, *p*_2_, …, *p*_*N*_ be the capture probabilities of the *N* individuals. The distribution of *X*_*i*_ given *p*_*i*_ follows a binomial distribution with a total number of trials *T* and a probability of success *p*_*i*_, so we write [*X*_*i*_|*p*_*i*_]∼ Binomial(*T*, *p*_*i*_). We assume *p*_1_, *p*_2_, …, *p*_*N*_ are independent and identically distributed random variables with distribution function *g*(*p*; ***θ***) with unknown parameter vector ***θ***. Here, *g*(*p*; ***θ***) is a probability density function that has support [0, 1] when *p* is continuous. We use the following capture probability models for heterogeneity: (a) beta distribution: *g*(*p*; ***θ***) = *g*(*p*; *α*, *β*) where *α* and *β* are parameters and (b) logit-normal distribution: *g*(*p*; ***θ***) = *g*(*p*; *μ*, *σ*) with parameters *μ* and *σ*.

Missing from capture-recapture data is *f*_0_, the number of individuals who are never captured during the *T* sampling occasions. Since *f*_*x*_ is the number of individuals captured *x* times, we only know *f*_*x*_ for *x* = 1, 2, …, *T*, and *f*_0_ is unknown. With this notation, the number of individuals captured at least once can be expressed as $n=\sum_{x=1}^{T}f_{x}$. If the probability density function *g*(*p*; ***θ***) of *p* is known, then the probability that a randomly selected individual will be captured *x* times can be expressed as πg(x)=∫01(Tx)px(1-p)(T-x)g(p;θ)dp.

It is clear that *f*_0_ = *N* − *n* and that ***f*** = (*f*_0_, *f*_1_, *f*_2_, …, *f*_*T*_)′ follows a multinomial distribution with (*T* + 1) cells and the corresponding cell probabilities are ***π***_***g***_ = (*π*_*g*_(0), *π*_*g*_(1), …, *π*_*g*_(*T*))′, where *π*_*g*_(*x*) is the probability of an individual sighted *x* times. More precisely, with a total population size of *N* and *T* sampling times, the distribution of the random vector of observed frequencies ***f*** is as follows [***f***]∼ Multinomial_*T*+1_(*N*, ***π***_***g***_). However, the multinomial distribution of ***f*** cannot be determined since *f*_0_ is unknown. Instead, we consider the distribution of the observable frequencies by conditioning on the total number of individuals captured, *n*. We denote the observable frequencies by ***f***^***c***^ = (*f*_1_, *f*_2_, …, *f*_*T*_)′, and write $\pi_{g}^{c}={\left(\pi_{g}^{c}\left(1\right),\pi_{g}^{c}\left(2\right),\ldots ,\pi_{g}^{c}\left(T\right)\right)}^{\prime }$ where $\pi_{g}^{c}\left(x\right)$ denotes the probability that an individual is sighted *x* times conditional on the individual sighted at least once. The conditional cell probabilities $\pi_{g}^{c}$ are calculated based on the unconditional probabilities ***π***_***g***_ as follows $\pi_{g}^{c}\left(x\right)=\pi_{g}\left(x\right)/\left\{1-\pi_{g}\left(0\right)\right\},x=1,2,\ldots ,T.$ The conditional distribution of ***f***^***c***^ given *n* is a multinomial distribution with a total of *n* trials and *T* cell probabilities $\pi_{g}^{c}$, that is, [***f***^***c***^|*n*]∼ Multinomial${}_{T}\left(n,\pi_{g}^{c}\right)$. The number of individuals *n* that are captured at least once follows a binomial distribution with a total of *N* trials and a probability of success 1 − *π*_*g*_(0). Thus, [*n*]∼ Binomial(*N*, 1 − *π*_*g*_(0)). Finally, it follows from the above that the distribution of ***f*** can be expressed as [***f***] = [***f***^*c*^|*n*][*n*].

The full likelihood of *N* and the unknown parameters can be written as *L*(***θ***, *N*; ***f***) ∝ [***f***] = [***f***^***c***^|*n*][*n*], which can be used to estimate *N* and ***θ*** simultaneously. If we condition on *n*, then *N* can be removed from the full likelihood and the resulting conditional likelihood for ***θ*** can be expressed as *L*^*c*^(***θ***; ***f***^***c***^) ∝ [***f***^***c***^|*n*]. More specifically, the distribution of [***f***^***c***^|*n*] is multinomial with probability mass function [fc|n]=(nf1,f2,…,ft)πgc(1)f1πgc(2)f2…πgc(T)fT. Here, elements of $\pi_{g}^{c}\left(x\right)$ are computed based on *π*_*g*_(*x*) and *π*_*g*_(0) where *π*_*g*_(*x*) is dependent on the underlying capture probability distribution.

To use the conditional likelihood to estimate the population size *N*, we first find the maximizer $\hat{\theta }$ of the conditional likelihood. With this estimated value of ***θ***, the distribution function $g(p;\hat{\theta })$ is available and the probability *π*_*g*_(0) can then be estimated. It follows that the population size *N* can be estimated using a Horvitz-Thompson type estimator [11] as $\hat{N}=n/\left\{1-{\hat{\pi }}_{g}\left(0\right)\right\}.$ This conditional likelihood method and the full likelihood method are asymptotically equivalent [3]. Therefore, they often give similar results when the number of individuals captured at least once (*n*) is large.

We note that all simulations in Section 3 and analyses of the white stork data in Section 4 were done using R software [18].

### The minimum chi-square method

Minimum chi-square estimation has a long history in statistics; see, e.g., [19, 20]. We now discuss this method and extend it to handle population size estimation with multi-occasion closed-population capture-recapture data.

Suppose *Y* is a multinomial random variable supported on {*y*_1_, *y*_2_, …, *y*_*K*_} with probability mass function *π*(*y*_*i*_; ***θ***) where *y*_*i*_’s are fixed constants, *K* ≥ 2 is a known integer, *π*(*y*_*i*_; ***θ***)>0 and ∑*π*(*y*_*i*_, ***θ***) = 1, and ***θ*** is a *d*-dimensional parameter vector. Let $\theta_{t}\in R^{d}$ denote the true but unknown value of the parameter vector. For a given value $\theta \in R^{d}$, consider testing the null hypothesis *H*_0_: ***θ***_***t***_ = ***θ*** with a random sample of *n* observations of *Y*. Let *O*_*i*_ denote the number of times *y*_*i*_ appears in the sample. Then, *O*_*i*_ is the observed value and *E*_*i*_(***θ***) = *nπ*(*y*_*i*_; ***θ***) is the expected value under *H*_0_. The Pearson chi-square statistic for the null hypothesis is given by

$$\begin{array}{c} X^{2}\left(\theta \right)=\sum_{i=1}^{K}\frac{{\left(O_{i}-E_{i}\left(\theta \right)\right)}^{2}}{E_{i}\left(\theta \right)}. \end{array}$$ (1)

Under *H*_0_, *X*^2^(***θ***) has an asymptotic chi-square distribution with (*K* − 1) degrees of freedom, so to test the null hypothesis we simply compare the observed value of the *X*^2^(***θ***) statistic with a chosen *χ*^2^ critical value. Those values of ***θ*** not rejected by the test form a region which [20] called the *consonance region* for ***θ***_***t***_. Denote by $R_{1-\alpha }$ the 100(1—*α*)% consonance region for ***θ***_***t***_. Then,

$$\begin{array}{c} R_{1-\alpha }=\left\{\theta \in R^{d}:X^{2}\left(\theta \right)\leq \chi_{\alpha ,K-1}^{2}\right\}, \end{array}$$ (2)

where $\chi_{\alpha ,K-1}^{2}$ is the (1 − *α*)th quantile of the $\chi_{K-1}^{2}$ random variable. This concept was first introduced by [21] who were more interested in how consonant the data are with the probability model *π*(*y*_*i*_; ***θ***) than estimating the unknown parameter vector ***θ***. We will also use the name consonance region to differentiate this region from other types of confidence regions. However, since our main interest is in parameter estimation, we will continue to call the quantity 100(1 − *α*)% associated with the consonance region its *confidence level*.

We now compare the consonance region (2) with the classical confidence region based on the asymptotic distribution of a maximum likelihood estimator for ***θ***_***t***_. The following are three key comparisons.

(i) The construction of the consonance region (2) does not require a point estimator. It is obtained by inverting the Pearson chi-square test in that it consists of ***θ*** values not rejected by the test at level-*α*. Apart from the Pearson chi-square test, other goodness-of-fit tests may also be used to derive consonance regions. For example, in [20], the Anderson-Darling test was also used to derive consonance regions. For discrete data such as the capture-recapture data, the chi-square test is a preferred natural choice.
(ii)The classical confidence region can be constructed for any confidence level (1 − *α*) ∈ (0, 1). For the consonance region, there is a lower bound on the confidence level; consonance regions with levels below this bound are empty. To see this, let $\tilde{\theta }={\text{argmin}}_{\theta }\left\{X^{2}\left(\theta \right)\right\},$ and let α* = P $\left(\chi_{K-1}^{2}\geq X^{2}\left(\tilde{\theta }\right)\right)$. Then, $X^{2}\left(\tilde{\theta }\right)$ is the (1 − α*)th quantile of the $\chi_{K-1}^{2}$ random variable. A consonance region with a confidence level (1 − *α*) < (1 − α*) is empty because there are no ***θ*** values satisfying $X^{2}\left(\theta \right)\leq \chi_{\alpha ,K-1}^{2}$ as the smallest *X*^2^(***θ***) value is $X^{2}\left(\tilde{\theta }\right)=\chi_{\alpha^{*},K-1}^{2}$, which is greater than $\chi_{\alpha ,K-1}^{2}$ because *α* > α*.
  This lower bound, as [20] pointed out, is not a cause for alarm and should be viewed as useful information. For example, suppose (1 − α*) = 0.91 so that a 90% consonance region is unavailable. This tells us that no model can pass the goodness-fit-test at the 10% level and this information is worth knowing. We note that a consonance region with a confidence level that happens to be below the bound derived from a particular sample is still valid; if the assumptions are all correct it may not be empty for the next sample and it will capture the true value 100(1 − *α*)% of the time when it is constructed repeatedly using independent random samples. When it is empty, it informs us that it is one of the 100*α*% times where the consonance region does not capture the true value. This is an advantage as none of the classical confidence regions that do not contain the true value would identify themselves as such.
Classical confidence regions based on the asymptotic distribution of a point estimator are nested in that if (1 − *α*_1_) < (1 − *α*_2_), then a region with confidence level (1 − *α*_1_) is fully contained by one with confidence level (1 − *α*_2_). The point estimate is the only point in the intersection of all confidence regions with confidence level (1 − *α*)>0. Consonance regions are also nested.

To extend the method of [20] to estimate *N*_*t*_, the true value of *N*, we assume that *g*(*p*; ***θ***) is correctly specified although the true value of its parameter ***θ***_***t***_ is unknown. Let *π*(*i*; ***θ***) be the marginal probability that a randomly selected individual from the population will be sighted exactly *i* times. Let *G*_*i*_ = {*j*: *X*_*j*_ = *i*} for *i* = 0, 1, …, *T*. Then, (a) *G*_*i*_ represents the group of individuals that have been observed on exactly *i* of the *T* occasions and (b) the *G*_*i*_ form a partition of the population. Let *O*_*i*_ denote the number of individuals in *G*_*i*_. Then, (b) implies *O*_0_ + *O*_1_ + … + *O*_*T*_ = *N*_*t*_. In a capture-recapture experiment, we do not know the number of individuals in *G*_0_ and thus *O*_0_ is not available. But if *N*_*t*_ is given, we can compute *O*_0_ by using the above equation $O_{0}=N_{t}-\sum_{i=1}^{T}O_{i}.$

Now consider testing the hypothesis *H*_0_: *N*_*t*_ = *N* versus *H*_1_: *N*_*t*_ ≠ *N* using the observed *O*_1_, *O*_2_, …, *O*_*T*_. For the time being, assume all *O*_*i*_ ≥ 5 and *T* > *d*, where *d* is the number of parameters for the underlying capture probability distribution *g*(*p*; ***θ***). Possible violations of these assumptions will be addressed in the simulation study section. Define

$$\begin{array}{c} X^{2}\left(N,\theta \right)=\sum_{i=0}^{T}\frac{{\left(O_{i}-E_{i}\left(\theta \right)\right)}^{2}}{E_{i}\left(\theta \right)}, \end{array}$$

where *E*_*i*_(***θ***) = *Nπ*(*i*; ***θ***) and $O_{0}=N-\sum_{i=1}^{T}O_{i}$. For a given *N*, let ${\tilde{\theta }}_{N}={\text{argmin}}_{\theta }\left\{X^{2}\left(N,\theta \right)\right\}.$ We define *the partial minimum chi-square statistic*,

$$\begin{array}{c} X^{2}\left(N,{\tilde{\theta }}_{N}\right)=\sum_{i=0}^{T}\frac{{\left(O_{i}-E_{i}\left({\tilde{\theta }}_{N}\right)\right)}^{2}}{E_{i}\left({\tilde{\theta }}_{N}\right)}, \end{array}$$ (3)

which has an asymptotic chi-square distribution with (*T* + 1) − 1 − *d* = *T* − *d* degrees of freedom under *H*_0_. That the chi-square statistic (3) defined by ${\tilde{\theta }}_{N}$ has degrees of freedom *T* − *d*, instead of (*T* + 1) − 1, was first shown in [22]. See [23] for an alternative estimator to ${\tilde{\theta }}_{N}$ that also leads to *T* − *d* degrees of freedom for the *X*^2^ statistic. It follows from [20] that a 100(1 − *α*)% consonance set for *N*_*t*_ is

$$\begin{array}{c} R_{1-\alpha }^{N}=\left\{N\in N:X^{2}\left(N,{\tilde{\theta }}_{N}\right)\leq \chi_{\alpha ,T-d}^{2}\right\}, \end{array}$$ (4)

where $\chi_{\alpha ,T-d}^{2}$ is the (1 − *α*)th quantile of the $\chi_{T-d}^{2}$ random variable. For point estimation of *N*_*t*_, let

$$\begin{array}{c} \left(\hat{N},\hat{\theta }\right)=\underset{\left(N,\theta \right)}{\text{argmin}}\left\{X^{2}\left(N,\theta \right)\right\}. \end{array}$$

Then $\hat{N}\in R_{1-\alpha }^{N}$ so long as $R_{1-\alpha }^{N}$ is not empty, regardless of the confidence level (1 − *α*). To see this, note that $X^{2}\left(\hat{N},\hat{\theta }\right)$ is the smallest value of *X*^2^(*N*, ***θ***). If an $R_{1-\alpha }^{N}$ is not empty, then there exists one pair $\left(N,{\tilde{\theta }}_{N}\right)$ such that $X^{2}\left(N,{\tilde{\theta }}_{N}\right)\leq \chi_{\alpha ,T-d}^{2}$. Since $X^{2}\left(\hat{N},\hat{\theta }\right)\leq X^{2}\left(N,{\tilde{\theta }}_{N}\right)$, we have $X^{2}\left(\hat{N},\hat{\theta }\right)\leq \chi_{\alpha ,T-d}^{2}$ and thus $\hat{N}\in R_{1-\alpha }^{N}$. This also implies that $\hat{N}$ is the only point in the intersection of all non-empty consonance sets for *N*_*t*_. By point (iii) in the comparison of consonance regions and classical confidence regions in the previous section, we see that $\hat{N}$ corresponds to the point estimator for *N*_*t*_. Hence, we use $\hat{N}$ as a point estimator for *N*_*t*_ and call it the *minimum chi- square estimator* of the population size. The theoretical properties of $\hat{N}$ are difficult to obtain and its variance is presently not available. Fortunately, the associated consonance set $R_{1-\alpha }^{N}$ is available which reduces the need for its standard error.

To construct a consonance region for the unknown parameter vector ***θ***_***t***_, consider testing the hypothesis *H*_0_: ***θ***_***t***_ = ***θ*** and *H*_1_: ***θ***_***t***_ ≠ ***θ*** using the chi-square statistic

$$\begin{array}{c} X^{2}\left(N_{t},\theta \right)=\sum_{i=0}^{T}\frac{{\left(O_{i}-E_{i}\left(\theta \right)\right)}^{2}}{E_{i}\left(\theta \right)} \end{array}$$

where *E*_*i*_(*θ*) = *Nπ*(*i*; ***θ***), *N*_*t*_ is the true population size and $O_{0}=N_{t}-\sum_{i=1}^{T}O_{i}$. Under *H*_0_, *X*^2^(*N*_*t*_, ***θ***) has a chi-square distribution with *T* degrees of freedom. It follows from [20] that a 100(1 − *α*)% consonance region for ***θ***_***t***_ is

$$\begin{array}{c} \left\{\theta \in R^{d}:X^{2}\left(N_{t},\theta \right)\leq \chi_{\alpha ,T}^{2}\right\} \end{array}$$ (5)

where $\chi_{\alpha ,T}^{2}$ is the (1 − *α*) quantile of the $\chi_{T}^{2}$ random variable. Since *N*_*t*_ is unknown, we replace it with $N_{\theta }={\text{argmin}}_{N}\left\{X^{2}\left(N,\theta \right)\right\}$ and replace (5) with

$$\begin{array}{c} R_{1-\alpha }^{\theta }=\left\{\theta \in R^{d}:X^{2}\left(N_{\theta },\theta \right)\leq \chi_{\alpha ,T}^{2}\right\}. \end{array}$$ (6)

Region $R_{1-\alpha }^{\theta }$ in (6) contains the region in (5) because *X*^2^(*N*_*θ*_, ***θ***) ≤ *X*^2^(*N*_*t*_, ***θ***). Hence, $R_{1-\alpha }^{\theta }$ is a conservative 100(1 − *α*)% consonance region for ***θ***_***t***_ as its coverage level is more than 100(1 − *α*)%. Simulation results show that its coverage level is close to 100(1 − *α*)% in many applications. Following the same argument used to show that $\hat{N}$ is in all non-empty consonance sets $R_{1-\alpha }^{\theta }$, we can show that $\hat{\theta }$ in $\left(\hat{N},\hat{\theta }\right)={\text{argmin}}_{\left(N,\theta \right)}\left\{X^{2}\left(N,\theta \right)\right\}$ is in all non-empty $R_{1-\alpha }^{\theta }$. This implies $\hat{\theta }$ is the “center” of each $R_{1-\alpha }^{\theta }$ and justifies its use as a point estimator for ***θ***_***t***_. We call $\hat{\theta }$ the minimum chi-square estimator for ***θ***_***t***_.

Finally, we use the chi-square statistic $X^{2}\left(\hat{N},\hat{\theta }\right)$ as a measure of goodness-of-fit for the heterogeneity model *g*(*p*; ***θ***). It measures how consistent the particular combination of $\hat{N}$ and $g(p;\hat{\theta })$ is with the observed data *O*_1_, *O*_2_, …, *O*_*T*_. Since $N=\hat{N}$ represents the most favourable *N* value under which to evaluate the goodness-of-fit of *g*(*p*; ***θ***) (in the sense that $X^{2}\left(\hat{N},\hat{\theta }\right)$ is the smallest possible value), the use of this statistic as a goodness-of-fit measure for *g*(*p*; ***θ***) is favourable to the model. To test the null hypothesis that the true heterogeneity model is $g(p;\hat{\theta })$ with $X^{2}\left(\hat{N},\hat{\theta }\right)$, a reasonable calibration of its limiting distribution is $\chi_{T-d}^{2}$ since under the null hypothesis $X^{2}\left(N_{t},\hat{\theta }\right)∼\chi_{T-d}^{2}$ and $\hat{N}$ estimates *N*_*t*_. If we use this limiting distribution and thus the *p*-value *p* = P $\left(\chi_{T-d}^{2}\geq X^{2}\left(\hat{N},\hat{\theta }\right)\right)$ for testing, then the type-I error may be slightly lower than the significance level *α*. This is because $X^{2}\left(\hat{N},\hat{\theta }\right)\leq X^{2}\left(N_{t},\hat{\theta }\right)$, and thus the *p*-value given above is larger than it would be if *N*_*t*_ had been known which leads to fewer rejections under *H*_0_.

### Bootstrap techniques

The empirical bootstrap is a commonly used statistical resampling technique, developed by [24], to estimate the variation of point estimates without making strong distributional assumptions. The key idea of bootstrapping is to make inference about a population based on the sample data, which can be modelled by resampling the sample data and performing inference about a sample from resampled data. It is now widely recognized as an adequate variance estimation method for capture-recapture studies [25]. In this section, we will discuss two different bootstrap methods, one is resampling from the same estimated distribution, which we refer to as the parametric bootstrap, and another is resampling of individuals from the whole population, which we refer to as the bootstrap of individuals. Bootstrap of individuals is similar to one of the methods discussed in [25]; however, they were concerned with constant capture probability. These two methods are analogous to the third method and the first method, respectively, in [26]. Rather than providing an estimate of variance, we focus on constructing a confidence interval for estimated population size. A parametric bootstrap approach was adopted by [27] based on maximum likelihood estimation to construct an interval estimate of the population size. We study the performance of the two bootstrap methods for constructing confidence intervals of the estimated population size *N* under minimum chi-square estimation through a simulation study. A comparison of confidence intervals based on the proposed bootstrap techniques for the white stork population size is presented in the application section. The accuracy of the consonance region such as (4) depends on the accuracy of the *χ*^2^ approximation to the final sample distribution of $X^{2}\left(N,{\tilde{\theta }}_{N}\right)$ which may be poor when the sample size is small. The bootstrap methodology provides an alternative means of interval estimation in such situations.

#### Parametric bootstrap

To obtain a parametric bootstrapped confidence interval with multi-occasion capture-recapture data, we first make an assumption about the underlying capture probability model, say we assume it is a beta distribution, without specifying its parameter vector ***θ***. We then estimate the population size *N* and ***θ*** using the data through either the minimum chi-square method or likelihood based methods. Let $\left(\hat{N},\hat{\theta }\right)$ be the estimated parameter values. We now have an (estimated) population of size $\hat{N}$ with a capture probability distribution fully specified by $\hat{\theta }$. This is our resampling distribution from which we generate a large number of bootstrap samples, say *m* samples. Here, each bootstrap sample is a multi-occasion capture-recapture data set with the same number of occasions *T* as the original data. Each sample is obtained by first generating a random sample of size $\hat{N}$ (integer) from the capture probability distribution, say $\left\{p_{1},p_{2},\ldots ,p_{\hat{N}}\right\}$ and then generating $\hat{N}$ binomial random numbers *B*_*i*_∼ Binomial(*T*, *p*_*i*_) for $i=1,2,\ldots ,\hat{N}$. The *p*_*i*_ represents the capture probability of the *i*^th^ individual in the population and *B*_*i*_ represents the number of times it is observed during the *T* occasions. From these *m* samples we obtain *m* bootstrap estimates of *N*, say $\left\{N_{1}^{⋆},N_{2}^{⋆},\ldots ,N_{m}^{⋆}\right\}$. Finally, we obtain a 95% parametric bootstrapped confidence interval for the population size *N* which is defined by the 2.5^th^ and 97.5^th^ percentiles of these estimates.

#### Bootstrap of individuals

An alternative to the above parametric bootstrap is to bootstrap the individuals (see [7]) as follows. With a set of multi-occasion capture-recapture data ***f***^***c***^ = (*f*_1_, *f*_2_, …, *f*_*T*_)′, we again first estimate the population size under an assumption about the capture probability distribution. Let $\hat{N}$ be the estimated integer value. We now have an (estimated) population of size $\hat{N}$ consisting of individuals in the data set and those that were never observed. Let *n* denote the total number of individuals that have been observed at least once. Then, the number of individuals never captured may be estimated by ${\hat{f}}_{0}=\hat{N}-n$. The estimated population can now be characterized by $f={\left({\hat{f}}_{0},f_{1},\ldots ,f_{T}\right)}^{\prime }$, that is, the population contains ${\hat{f}}_{0}$ 0’s, *f*_1_ 1’s, and so on. Next, we take a random sample of size $\hat{N}$ from this population with replacement. Since each individual is replaced before the next individual is drawn, some individuals may appear more than once, and others may never appear at all. Each sample is a bootstrap replicate of the original capture-recapture data. We repeat this process *m* times, obtaining *m* sets of such data with which we compute *m* bootstrap estimates of *N*, say $\left\{N_{1}^{⋆},N_{2}^{⋆},\ldots ,N_{m}^{⋆}\right\}$. We then use the 2.5^th^ and 97.5^th^ percentiles of these $N_{i}^{⋆}$ to obtain the 95% individual bootstrapped confidence interval for *N*. Compared with the parametric bootstrap, this bootstrap of individuals is less dependent on the capture probability model assumption as it does not use this assumption in the resampling step of the operation.

Numerical integration was used to obtain capture probability estimates *π*_*g*_(*x*). Further, to obtain consonance regions for model parameters *θ* and *N* we performed a grid search. More details are provided in the S1 File and examples of code are provided on Github at https://github.com/ILR819/white_stork.

## Simulation studies

### Accuracy of the chi-square approximation

In real applications, the accuracy of the chi-square approximation to the finite sample distributions of chi-square statistics in (1) and (3) depends on the size of *n* and *N*, respectively. For (1), it is well known that when *n* is sufficiently large so that the observed cell counts *O*_*i*_ are all more than 5, the approximation is good. For (3), the accuracy of the approximation depends on not only *N* but also the number of parameters estimated *d* and the number of categories or cells *T* + 1 in the chi-square statistic, so it is important to examine the accuracy empirically for the combination of (*N*, *d*, *T*) that we are interested in before we use the asymptotic chi-square distribution to construct consonance intervals for the unknown *N*.

We now present a simulation study on the accuracy of the chi-square approximation for (3) for the case where *N* = 4000 and *T* = 10. These *N* and *T* values were chosen because of the prior information about *N* concerning the white stork population and the data available. For the capture probability distribution, we chose beta(1, 10) as simulation results show that it produces similar observed frequency vector ***f***^***c***^ to the white stork data. With the chosen *N*, *T* and beta(1,10), we first generate multi-occasion capture-recapture data as described in the parametric bootstrap, and then pretend the parameter values of the beta distribution are unknown and estimate them using beta(*α*, *β*) as the unknown capture probabililty distribution with the minimum chi-square estimator ${\tilde{\theta }}_{N}$. The resulting $X^{2}\left(N,{\tilde{\theta }}_{N}\right)$ value is a random observation of the partial minimum chi-square statistic (3). We repeated this process 200 times, obtaining 200 sets of simulated multi-occasion capture-recapture data and 200 random observations of the $X^{2}\left(N,{\tilde{\theta }}_{N}\right)$ statistic. These 200 observations form a random sample of size 200 from the null distribution of the partial minimum chi-square statistic $X^{2}\left(N,{\tilde{\theta }}_{N}\right)$. We then used this sample of size 200 to determine the accuracy of the chi-square approximation through QQ-plots which plot the sample quantiles against that of the corresponding quantiles of the asymptotic chi-square distribution. Recall that the degrees of freedom of the partial minimum chi-square statistic $X^{2}\left(N,{\tilde{\theta }}_{N}\right)$ in (3) equals the total number of cells *C* = *T* + 1 minus (*d* + 1) where *d* is the dimension of ***θ***. Here, in order to have a positive degrees of freedom and to ensure cell counts *O*_*i*_ are not too small, we pooled the data (aggregating counts for some sampling occasions) into a total of *C* = 4, 5, 6, 7 cells; see the white stork data analysis in the next section for examples of such aggregation. With *d* = 2 for the beta model, the degrees of freedom of the asymptotic chi-square distribution are *df* = 1, 2, 3, 4, respectively.

**Fig 1:**
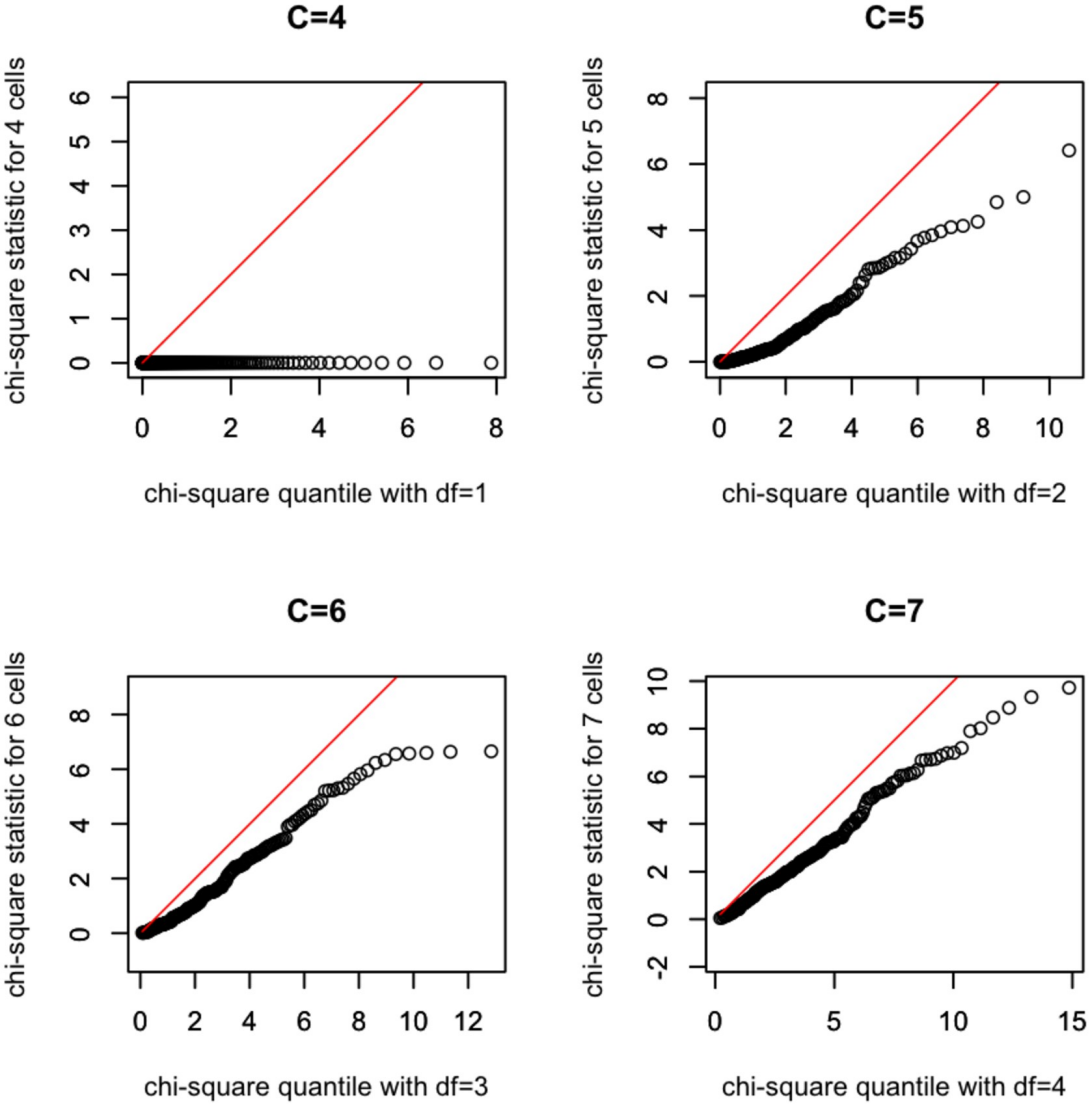
QQ-plots of a sample of *n* = 200 partial minimum chi-square statistic values versus quantiles of the asymptotic chi-square distribution for C = 4, C = 5, C = 6, and C = 7, respectively, where *N* = 4000, *T* = 10, and the capture probability distribution is beta. The red line in each plot is the *y* = *x* line.

Fig 1 shows the QQ-plots of the sample of 200 for the four cases. We see from the plots that for the present combination of *N*, *T* and capture probability distribution, the asymptotic chi-square approximation to the finite sample distribution of the partial minimum chi-square statistic $X^{2}\left(N,{\tilde{\theta }}_{N}\right)$ is not accurate. In particular, for *C* = 4, the statistic $X^{2}\left(N,{\tilde{\theta }}_{N}\right)$ has very small values, indicating the chi-square approximation is very poor. With more cells the approximation becomes better but still not accurate enough for constructing consonance intervals for *N*. Nevertheless, we note that the simulated quantiles of $X^{2}\left(N,{\tilde{\theta }}_{N}\right)$ tend to be smaller than the corresponding chi-square quantiles when the underlying capture probability distribution is correctly specified, regardless the total number of cells *C*. Thus this statistic is still useful for testing whether or not the capture probability distribution used is correct; if an observed value of the statistic exceeds say the 0.95th quantile of the asymptotic chi-square distribution, then the capture probability model should be rejected at 5% level based on this empirical finding. This test is conservative in that its type-I error is less than 5% but it may not be very powerful against misspecified capture probability models. Nevertheless, in the absence of other tests for the capture probability model, we will apply this test when analyzing the white stork data.

Finally, the poor accuracy of the chi-square approximation for the present combination of *N*, *T* and capture probability model does not invalidate the minimum chi-square method as a method of point estimation. It simply indicates the asymptotic chi-square distribution cannot be used to calibrate the consonance interval for this particular combination. Further, there are many situations where the chi-square approximation has been found to be accurate when the population size *N* is large. See the S1 File for examples where the chi-square approximation is accurate.

### Bootstrap techniques for interval estimation

**Fig 2:**
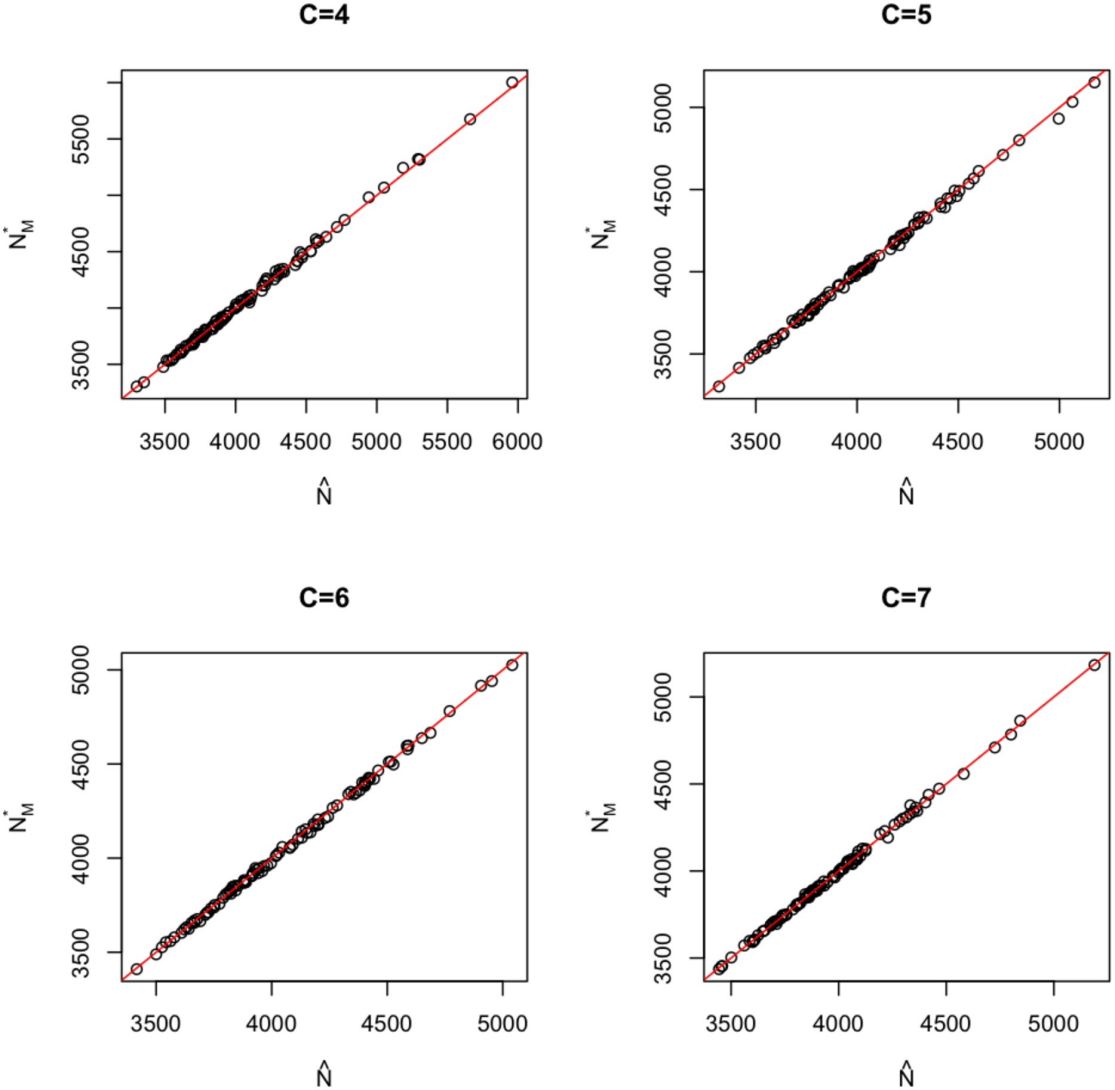
The geometry of the bootstrap confidence intervals: Plots of the 100 point estimates ${\hat{N}}_{i}$ versus the “centre-point” of the confidence interval $N_{i,M}^{⋆}$ for C = 4, C = 5, C = 6, and C = 7 where *N* = 4000, *T* = 10 and the capture probability distribution is beta. The red line is the *y* = *x* line.

Since the chi-square approximation cannot be used to compute confidence intervals for the above combination of *N*, *T* and capture probability model, we use the bootstrap instead. To see that the bootstrap is effective, we first generated 100 sets of multi-occasion capture-recapture data and used these to obtain 100 pairs of estimated parameters values $\{\left({\hat{N}}_{1},{\hat{\theta }}_{1}\right)$, $\left({\hat{N}}_{2},{\hat{\theta }}_{2}\right)$, …, $\left({\hat{N}}_{100},{\hat{\theta }}_{100})\right\}$. Each pair of estimated parameters $\left({\hat{N}}_{i},{\hat{\theta }}_{i}\right)$ defines an estimated population from which we resample, either through parametric bootstrap or bootstrap of individuals, to generate 1000 sets of capture history data with which we produce 1000 estimates of the population size $N_{i,j}^{⋆}$ for *j* = 1, 2, …, 1000. We then constructed a 95% confidence interval using percentiles of $N_{i,j}^{⋆}$ as described in Section 2.3, resulting in 100 different bootstrapped intervals $\{[N_{1,L}^{⋆},$ $N_{1,U}^{⋆}],$ $[N_{2,L}^{⋆},$ $N_{2,U}^{⋆}],$ …, $[N_{100,L}^{⋆},$ $N_{100,U}^{⋆}]\}$. To see these are reasonable bootstrap intervals, we plotted the point estimate ${\hat{N}}_{i}$ against the “centre-point” of the interval $[N_{i,L}^{⋆},$ $N_{i,U}^{⋆}]$ represented by the median of $\{N_{i,1}^{⋆},N_{i,2}^{⋆},\ldots ,N_{i,1000}^{⋆}$} which we denote with $N_{i,M}^{⋆}$. Fig 2 shows such plots of $\left({\hat{N}}_{i},N_{i,M}^{⋆}\right)$ when the number of cells equals to 4, 5, 6, or 7, respectively. The lines in each plot is the *y* = *x* line. We see that regardless of the number of cells, the $\left({\hat{N}}_{i},N_{i,M}^{⋆}\right)$ pairs are located around the *y* = *x* line indicating that the point estimate ${\hat{N}}_{i}$ is in the centre of the bootstrap confidence interval. Also, among the 100 confidence intervals for each *C* value, the percentage of the intervals containing the true population size *N* = 4000 is around 95% when the capture probability distribution is correctly specified, very close to the confidence level of 95%. We did the above simulation and plots for other combinations of *N*, *T* and capture probability distribution and obtained similar results.

**Fig 3:**
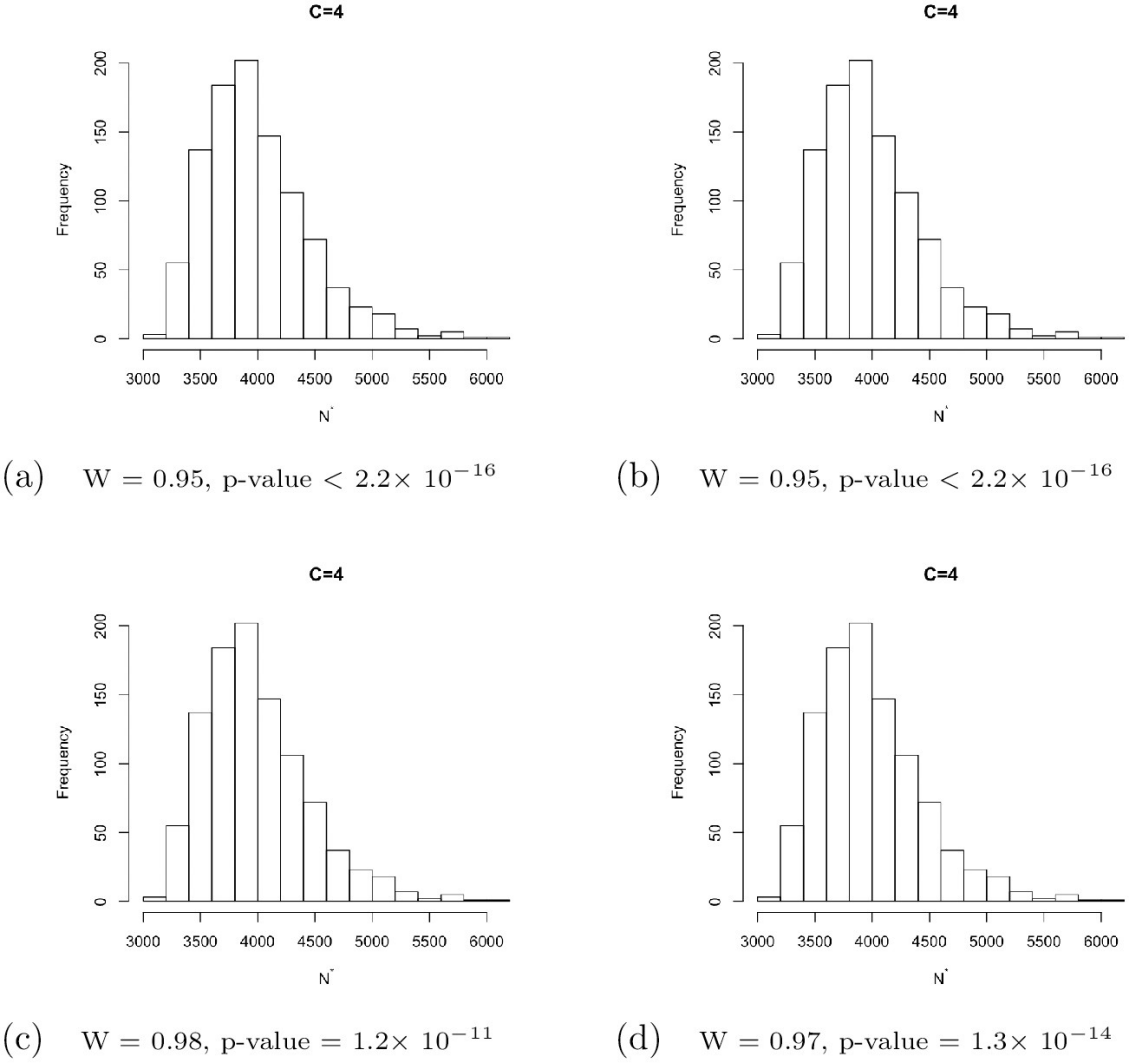
Histograms of samples of size 1000 bootstrap minimum chi-square estimates for the population size for C = 4, C = 5, C = 6, and C = 7, respectively, where *N* = 4000, *T* = 10 and the capture probability model is beta. Below the plots are the test statistics and the corresponding *p*-values of Shapiro-Wilk test for the null hypothesis that the bootstrap estimates are normally distributed.

Fig 3 shows the histograms of randomly selected sets of bootstrap estimates $\{N_{i,1}^{⋆},N_{i,2}^{⋆},\ldots ,N_{i,1000}^{⋆}$} for the 4-cells, 5-cells, 6-cells, and 7-cells cases, respectively. It is clear that the shape of the histogram for each case is non-normal. The test statistic and the corresponding *p*-value of Shapiro-Wilk normality test underneath each histogram further support this observation. This is consistent with the skewness of the distribution of population size estimators for all proposed methods in the capture-recapture literature; see [28]. Because of this, normal based confidence intervals cannot be used. When the asymptotic chi-square approximation is accurate, we can use the consonance set (4) as our confidence interval. When it is not, such as in our present case, we need to use the above bootstrap based confidence intervals instead.

### Bias in population size estimation

Through a thorough simulation study that varied population size *N*, *E*(*p*), and the generating capture probability distribution, we investigated robustness in terms of bias and root mean-square error of $\hat{N}$ (see Section 2.3 of the S1 File). In most cases, the minimum chi-square method outperforms the likelihood methods. In particular, when population size *N* is large and expected capture-probability *E*(*p*) is high, the minimum chi-square method outperforms the likelihood methods regardless of the number of cells used.

## Application to the white stork data

[12] conducted a study on the foraging strategies of white storks (*Ciconia ciconia*) as a closed population in southwestern Spain. They investigated individual foraging specialization. The data were collected by two observers within the white stork’s main wintering area in southwestern Spain. At each sampling occasion, they recorded a “1” if marked storks were observed at rice fields, a “2” if marked storks were observed at dumps, and a “0” if marked storks were not detected in a particular occasion. In total, 1684 different individuals were banded on 80 sampling occasions during the study period lasting 80 days. [12] found apparent survival rate was close to 1 during the winter study concluding that a closure assumption was valid. Due to the large study area and the large number of sampling occasions, the data are sparse. So we aggregated the daily white storks capture history data to reduce the number of sampling occasions from *T* = 80 to *T* = 10. This was done by pooling, for example, the first 8 days’ observations together to form the observation for the first (aggregated) sampling occasion; a white stork is recorded as “captured” on this sampling occasion if it was captured at least once in the first 8 days, and recorded as “not captured” otherwise. After the aggregation, the observed frequency vector **f**^**c**^ = {1021, 420, 166, 50, 20, 6, 1, 0, 0, 0}. To avoid zero count cells and small cell counts, we further aggregated the counts in the right tail of **f**^**c**^ and considered the following four cases when implementing the minimum chi-square method:

1. 4-cells case (*C* = 4): {1021, 420, 243}
2. 5-cells case (*C* = 5): {1021, 420, 166, 77}
3. 6-cells case (*C* = 6): {1021, 420, 166, 50, 27}
4. 7-cells case (*C* = 7): {1021, 420, 166, 50, 20, 7}

The 4-cell case, for example, includes a cell for unobserved individuals whose count is unknown.

An assumption often made in the modelling of capture-recapture data is that of homogeneity in the capture probability. The homogeneity model postulates that the capture probability *p* does not vary from one individual to another and over sampling occasions. Under this model, *g*(*p*; *θ*) is a point mass of 1 at *p*, and *π*_*g*_(*x*) is πg(x)=(Tx)px(1-p)T-x. Under this assumption, we computed the estimated *N* by using the minimum chi-square method, the full likelihood method, and the conditional likelihood method (Table 1). The full likelihood method gives an estimated population size of 2434 white storks and the capture probability *p* is estimated to be 0.11. The conditional likelihood method gives an estimate of 2433 with an estimated capture probability of 0.11 as well. For the minimum chi-square method, estimate of the population size gradually decreases from 2500 to 2344 as the number of cells increases which are largely in agreement with that given by the full and conditional likelihood methods. Moreover, the minimum chi-square statistics as a measure of goodness-of-fit is large relative to the corresponding chi-square distribution for all four cases. Consequently, the *p*-values are highly significant which suggests that heterogeneity in capture probability may exist and the point estimate obtained based on homogeneity assumption is not reliable. Note that this information is not available if we use the likelihood methods alone.

**Table 1:** Maximum full likelihood, maximum conditional likelihood and minimum chi-square estimates under the homogeneity assumption for capture probability. C is the number of cells after pooling counts in the right tail of **f**^**c**^, $\hat{N}$ is the estimated value of *N* and $\hat{p}$ is the estimated capture probability *p*. The chi-square statistic *X*^2^, its degree of freedom *df* and *p*-value summarize the minimum chi-square goodness-of-fit test for the homogeneity model for capture probabilities.

Method	Number of Cells	$\hat{N}$	$\hat{p}$	*X* ^2^	*df*	*p*-value
Full likelihood	NA	2434	0.11	−	−	−
Conditional likelihood	NA	2433	0.11	−	−	−
Minimum chi-square	C = 4	2500	0.11	29.001	2	<0.0001
	C = 5	2439	0.11	52.727	3	<0.0001
	C = 6	2375	0.11	91.577	4	<0.0001
	C = 7	2344	0.12	111.297	5	<0.0001

To take the heterogeneity in capture probabilities into consideration, we first consider modelling the heterogeneity using the beta distribution as it can take on many different shapes. The probability density function of the beta distribution is *g*(*p*; ***θ***) = *g*(*p*; *α*, *β*) = Γ(*α* + *β*)/{Γ(*α*)Γ(*β*)}*p*^*α*−1^(1 − *p*)^*β*−1^ where *α* > 0 and *β* > 0 are two shape parameters. Table 2 shows the results under the beta model for capture probability. The minimum chi-square estimate of the population size does not change very much with the C value indicating it is robust against the number of cells used. The small minimum chi-square statistics and the corresponding large *p*-values (close to 1) indicate that the beta model is appropriate for the capture probability. The minimum chi-square confidence intervals are also robust against the number of cells used. Both the minimum chi-square point estimate of *N* and the 95% CI are consistent with the full and conditional likelihood methods. To summarize, the minimum chi-square statistic shows that the heterogeneity in capture probability is present and the beta distribution is an appropriate model for the heterogeneity. It also provides a credible estimate of the white stork population size that is similar to the likelihood estimates and agrees with the census findings of 4000 [2].

**Table 2:** Maximum full likelihood, maximum conditional likelihood and minimum chi-square estimates under the beta model for capture probability. C is the number of cells after pooling counts in the right tail of **f**^**c**^ and $\hat{N}$ is the minimum chi-square point estimator of *N*. The chi-square statistic *X*^2^, its degree of freedom *df* and *p*-value summarize the minimum chi-square goodness-of-fit test for the beta model. Interval estimates and their widths using parametric bootstrap and bootstrap of individuals are also shown.

Method	Number of Cells	$\hat{N}$	*X* ^2^	*p*-value	95% CI^1^	Width^1^	95% CI^2^	Width^2^
Full	NA	4019	−	−	[3261, 5356]	2095	[3259, 5279]	2020
Conditional	NA	4000	−	−	[3298, 5481]	2183	[3243, 5464]	2221
Chi-square	C = 4	4033	<0.01	0.99	[3107, 6186]	3079	[3147, 6070]	2923
	C = 5	3852	0.23	0.89	[3109, 5358]	2249	[3171, 5219]	2048
	C = 6	4046	1.19	0.75	[3289, 5587]	2298	[3280, 5538]	2258
	C = 7	4036	1.20	0.88	[3309, 5617]	2308	[3290, 5682]	2392

^1^: Parametric bootstrap;

^2^: Bootstrap of individuals

We now use the logit-normal distribution to model the heterogeneity of the capture probability. Its density function is $g\left(p;\theta \right)=g\left(p;\mu ,\sigma \right)=1/\left(\sigma \sqrt{2\pi }\right)\cdot 1/\left\{p\left(1-p\right)\right\}exp(\left[-log\left\{p/\left(1-p\right)\right\}-\mu \right]/\left(2\sigma^{2}\right)),$ where *μ* is the mean and *σ* > 0 is the standard deviation. We estimated the white stork population size using the minimum chi-square method, and the full and conditional likelihood methods with this model (Table 3). The point estimates of the model parameters as well as the population size are quite stable for all cases. The relatively large *p*-values of the minimum chi-square method indicates the logit-normal model is also acceptable for the white stork data. For the minimum chi-square method, the confidence intervals become stable as the number of cells increases for both bootstrap techniques. When the number of cells *C* = 7, the two bootstrap intervals are only slightly wider than the ones given by the likelihood methods. The point estimates under the logit-normal case range from about 3350 to 3500, which are 500 to 600 individuals fewer than that under the beta model or the census findings. Most of the confidence intervals do not contain the number 4000, but they are narrower than the confidence intervals under the beta model.

**Table 3:** Maximum full likelihood, maximum conditional likelihood and minimum chi-square estimates under the logit-normal model for the capture probability. C is the number of cells after pooling the right tail of **f**^**c**^ and $\hat{N}$ is the point estimator of *N*. The chi-square statistic *X*^2^, its degree of freedom *df* and *p*-value summarize the minimum chi-square goodness-of-fit test for the logit-normal model. Interval estimates and their widths using parametric bootstrap and bootstrap of individuals are also shown.

Method	Number of Cells	$\hat{N}$	*X* ^2^	*p*-value	95% CI^1^	Width^1^	95% CI^2^	Width^2^
Full	NA	3355	−	−	[2995, 3834]	839	[2995, 3785]	790
Conditional	NA	3361	−	−	[2995, 3790]	795	[3011, 3777]	766
Chi-square	C = 4	3501	<0.01	0.99	[2987, 4259]	1272	[3002, 4257]	1255
	C = 5	3354	0.66	0.42	[2943, 3842]	899	[2990, 3882]	892
	C = 6	3407	1.01	0.80	[3055, 3901]	846	[3046, 3934]	888
	C = 7	3386	1.22	0.88	[3037, 3849]	812	[3029, 3833]	804

^1^: Parametric bootstrap;

^2^: Bootstrap of individuals

## Conclusion

We explored the minimum chi-square method for estimating a closed population size with multi-occasion capture-recapture data as an alternative method to likelihood based methods. The advantage of the minimum chi-square method is that it not only estimates the unknown population size but also performs goodness-of-fit test on the capture probability model. Further, the partial minimum chi-square statistic has an asymptotic chi-square distribution which can be easily used to construct consonance/confidence interval when the chi-square approximation is accurate. It is important to examine the accuracy of the chi-square approximation through simulations. When the accuracy is unsatisfactory, which may happen when the population is small, we use the bootstrap methods to construct confidence intervals.

In the white stork application, the estimated population size dropped by more than 10% for all methods when we changed the capture probability model from beta to logit-normal and yet both beta and logit-normal models seem to fit the data well. This raises the question of which estimate and which model we should trust. This is related to the nonidentifiability problem we noted in the introduction. While more work is needed to address this problem, through extensive simulation [29] showed that the minimum chi-square estimator $\hat{N}$ is robust against misspecification of the capture probability model *g*(*p*; ***θ***) for the case of a large population size of *N* = 10000 and an expected capture probability E(*p*) that is not too small; that is, we obtain good estimates of *N* even when the capture probability model is misspecified. In this case, the minimum chi-square method outperforms the existing likelihood based estimators in terms of bias and mean square error. The asymptotic chi-square distribution is also accurate and the associated consonance set $R_{1-\alpha }^{N}$ also performs well in terms of coverage accuracy. See the S1 File for some of the simulation results from [29]. Nevertheless, closed populations tend to be small (e.g. a confined population of 135 cottontail rabbit in [30]; an estimated closed population size of 173 of meadow vole in [2]). Thus the bootstrap approach to inference is necessary for these cases. But for population sizes at or above 10000, the consonance interval of the minimum chi-square method is a more natural interval estimate for the population size.

Our beta model based analysis of the white stork data provided supporting evidence to the existing estimate of *N* = 4000 obtained by the scientists through other means. Because the logit-normal model based estimates differ substantially from this number, we conclude that the capture probability of the white storks likely follows a beta distribution.

Finally, we note that the model we considered assumes only individual heterogeneity among capture probabilities. We did not consider the additional complexity of time-dependent individual heterogeneity in capture probability which would require restructuring the model. We leave this as an interesting future research direction.

## Supporting information

S1 File Simulation studies. Additional simulation studies of the accuracy and robustness of the minimum chi-square method.
(PDF)
