# Supplementary material for: Minimum chi-square method for estimating population size in capture-recapture experiments
Source: PLoS One. 2023 Oct 12;18(10):e0292622. doi: 10.1371/journal.pone.0292622 (PMC10569566; doi:10.1371/journal.pone.0292622)
Supplement: S1 File — Additional simulation studies of the accuracy and robustness of the minimum chi-square method. (PDF) [file pone.0292622.s001.pdf]

Supplementary Information for

Minimum chi-square method for estimating population size in capture-recapture experiments

Yuyan Zheng, Yongfei Mao, Min Tsao, Laura L.E. Cowen*

Department of Mathematics and Statistics, University of Victoria, Victoria, BC, Canada V8W 2Y2

*Corresponding Author Email: lcowen@uvic.ca

We present a simulation study on the performance of the minimum chi-square method for situations where the population size N is large ($N = 10,000$). We consider several scenarios with different number of sampling occasions and different capture probability distributions. We demonstrate that (i) the minimum chi-square estimator $\hat{N}$ is accurate in terms of both absolute bias and mean squared error in these scenarios, and (ii) the asymptotic chi-square approximation to the finite sample distribution of the partial minimum chi-square statistic defined in (3) of the main article is overall accurate. Additionally, we also compare the minimum chi-square method to the full and conditional likelihood methods in terms of robustness against misspecifications of capture probability models.

For computing the minimum chi-square estimate $\hat{N}$, we use a two-stage grid search strategy and a general purpose optimization algorithm 'L-BFGS-B' to find the global minimizer of the chi-square statistics (this minimizer is the estimate $\hat{N}$). The two-stage grid search is as follows. We start with a low initial value of N, say $N = 100$, and then increase N by 50 each time. For each N, we use 'L-BFGS-B' to find the corresponding value of the minimum chi-square statistic. This first stage of grid search leads to a narrow range of N values with small chi-square statistic values, say $(8000 - 50, 8000 + 50)$. Then, in the second stage of grid search, we compute the minimum chi-square statistic values for all N values in the range to find the value that gives the lowest chi-square statistic. This value is taken to be the global minimizer of the chi-square statistic and hence the minimum chi-square estimate $\hat{N}$. This two-stage strategy is based on our observation that the minimum chi-square statistic is usually a convex function of N, so its global minimum can be found by this two stage strategy. When there is no constraint on computation resources, we can simply remove the first-stage and find the global minimizer by trying all N values in a range of interest. Example

code can be accessed on Github at `https://github.com/ILR819/white_stork`.

1 Accuracy of the minimum chi-square method

In this section, we evaluate the accuracy of the minimum chi-square method when the population size N is large ($N = 10,000$). We first examine the accuracy of the minimum chi-square estimator $\hat{N}$ in terms of its bias and mean squared error (MSE). We then examine the accuracy of the asymptotic chi-square approximation to the finite sample distribution of the partial minimum chi-square statistic. In the simulation study, the number of sampling occasions considered are $T = 5, 10$. The distribution of the capture probability p is assumed to be Beta, and three Beta distributions with expected values $E(p) = 0.05, 0.10, 0.20$, representing populations with low to high capture probabilities, are considered. For each combination of T and capture probability distribution, we simulate the multi-stage capture-recapture process which leads to a set of simulated multi-stage capture-recapture data. We then apply the minimum chi-square method to the set of data to estimate N and compute the partial minimum chi-square statistic. We repeat this process 50 times, obtaining 50 observed values of $\hat{N}$ and 50 observed values of the partial minimum chi-square statistic. These values are then used to evaluate the accuracy of the minimum chi-square estimator and the asymptotic chi-square approximation.

1.1 Bias and MSE of the minimum chi-square estimator

Table S1 contains the bias, MSE and square root of the MSE of the minimum chi-square estimator $\hat{N}$ based on $C = 2, 3, 4, 5, 6$ cells for situations with $T = 5$ sampling occasions. We see that the 2-cell method has the highest bias and MSE and the 3-cell method has the lowest bias and MSE. This shows that the 2-cell method cannot make effective use of the multi-stage capture-recapture data when $T = 5$, and is thus not recommended. On the other hand, with $T = 5$, setting the number of cells to a relatively high number ($T = 5, 6$) may also be inappropriate. Considering that the exact population size is $N = 10,000$, except for the 2-cells method, the minimum chi-square estimator is quite accurate in terms of both bias and MSE when $E(p)$ is not too low.

Table S2 contains the bias, MSE and square root of the MSE for the minimum chi-square estimator based on $C = 2, 3, 4, 5, 6$ cells for situations with $T = 10$ sampling occasions. Again, we see that the 2-cell method has the highest bias and MSE, indicating that the 2-cell method cannot take full advantage of the multi-stage capture-recapture data with $T = 10$. We also see that while the 3-cells method is the best in terms of MSE, for the case of $E(p) = 0.05$, it has a higher bias than methods with more cells. When $E(p) \geq 0.10$, methods with 3 or more cells are approximately comparable in terms of bias and MSE, suggesting that when the number of sampling occasions T is large and the expected capture probability is not too low, the performance of the minimum chi-square estimator is not sensitive to the number of cells provided that it is more than 2.

Table S1. $T = 5$ sampling occasions: estimated bias and MSE of the minimum chi-square estimator with 2-cells, 3-cells, 4-cells, 5-cells and 6-cells based on simulated samples of size $n = 50$.

Sample Size	$E(p)$	Number of Cells	$bias(\hat{N})$	$MSE(\hat{N})$	$\sqrt{MSE(\hat{N})}$
50	0.05	2	3451.32	12 166 995.0	3488.12
50	0.05	3	495.53	472 025.8	687.04
50	0.05	4	524.51	6 031 063.0	2455.82
50	0.05	5	802.24	7 450 158.0	2729.50
49	0.05	6	774.23	7 519 929.0	2742.25
50	0.10	2	2161.55	4 846 747.0	2201.53
50	0.10	3	113.51	104 655.9	323.51
50	0.10	4	163.39	724 301.3	851.06
50	0.10	5	247.09	950 979.6	975.18
50	0.10	6	436.12	1 210 841.0	1100.38
50	0.20	2	1098.71	1 235 703.0	1111.62
50	0.20	3	27.84	11 765.8	108.47
50	0.20	4	73.80	80 664.2	284.01
50	0.20	5	35.58	49 941.6	223.48
50	0.20	6	49.96	46 991.7	216.78

Note that for the case of $E(p) = 0.05$ and 6-cells, our R code for computing the minimum chi-square estimate failed to converge in one of the 50 simulation runs, so for this case we only have a sample of 49 simulated values of the estimator.

Table S2. $T = 10$ sampling occasions: estimated bias and MSE of the minimum chi-square estimator with 2-cells, 3-cells, 4-cells, 5-cells and 6-cells based on simulated samples of size $n = 50$.

Sample Size	$E(p)$	Number of Cells	$bias(\hat{N})$	$MSE(\hat{N})$	$\sqrt{MSE(\hat{N})}$
50	0.05	2	3211.02	10 523 893.0	3244.06
50	0.05	3	419.41	266 232.3	515.98
50	0.05	4	253.42	1 670 340.0	1292.42
50	0.05	5	156.68	1 080 343.0	1039.40
50	0.05	6	143.65	992 230.3	996.11
50	0.10	2	1727.86	3 137 510.0	1771.30
50	0.10	3	45.90	22 341.2	149.47
50	0.10	4	87.32	134 713.9	367.03
50	0.10	5	53.02	85 881.0	293.05
50	0.10	6	48.38	77 156.4	277.77
50	0.20	2	531.23	290 536.0	539.01
50	0.20	3	6.83	1818.9	42.65
50	0.20	4	5.79	5531.0	74.37
50	0.20	5	9.56	3106.5	55.74
50	0.20	6	7.98	3065.5	55.37

We have considered other scenarios with a small number of sampling occasions ($T \leq 3$) and small expected capture probability ($E(p) < 0.02$). In such cases, the number of observed individuals in the capture-recapture experiments are very small, and all methods, including the minimum chi-square method, perform poorly. In general, for large populations of $N \geq 10,000$, the minimum chi-square method with $C \geq 3$ is accurate in terms of bias and MSE, provided that T and $E(p)$ are not too small.

1.2 Accuracy of the chi-square approximation

In Section 3 of the main article, we have noted that the asymptotic chi-square approximation to the finite sample distribution of the partial minimum chi-square statistic (3) is not accurate for the white stork population, likely due to the fact that the white stork population of roughly $N = 4000$ is not sufficiently large. In this subsection, we consider large populations of $N = 10,000$. Similar to the previous subsection, for each combination of $(T, E(p), C)$, we generate 50 sets of multi-stage capture-recapture data and compute the value of the partial minimum chi-square statistic for each set. This gives us a simulated random sample of $n = 50$ values of the statistic. We then generate a QQ-plot which plots this sample of 50 values against the quantiles of the asymptotic chi-square distribution to examine the accuracy of the asymptotic approximation. For brevity of presentation, the levels of the three factors that we will consider are $T = 5, 10$, $E(p) = 0.05, 0.10, 0.15, 0.20$, and $C = 4, 5, 6$, so there are $2 \times 4 \times 3 = 24$ QQ-plots to examine. The 24 QQ-plots are shown in Figures 1 to 6 where each figure contains four QQ-plots with different combinations of $(T, E(p))$ but the same C value so that degree of freedom of the underlying asymptotic Chi-square distribution (which depends only on C) is the same for all four QQ-plots.

Figure 1 shows the QQ-plots for samples of partial minimum chi-square statistic of 4-cells method at $T = 5$ against the exact quantiles of the chi-square distribution with 1 degree of freedom. Comparing these QQ-plots with the QQ-plot for the 4-cells method in Figure 1 of the main article, which is based on a population of size $N = 4000$, we see that the accuracy of the asymptotic chi-square approximation to the finite sample distribution of the partial minimum chi-square statistic has improved now that the population size is $N = 10,000$. There are still varying degrees of deviations from the theoretical chi-square distribution with 1 degree of freedom as the plots deviate from the line of $x = y$ at the upper tail, but the extent of such deviations are much smaller relative to those observed at $N = 4000$. Figure 2 contains the QQ-plots for 5-cells method at $T = 5$, and Figure 3 contains the QQ-plots for 6-cells method at $T = 5$. The asymptotic chi-square distributions corresponding to these two figures are of degrees of freedom 2 and 3, respectively. Again, we see similar improvements in the accuracy of the asymptotic chi-square approximation to the finite sample distribution of the partial minimum chi-square statistic. Figures 4, 5 and 6 contain the QQ-plots for different combinations of $(E(p), C)$ at $T = 10$. With 10 sampling occasions, we get more information about the underlying population, and the asymptotic chi-square approximation improves further. In this case, higher cell numbers seem to give better approximations, regardless the expected value of the capture probability.

2 Robustness comparison

In this section, we perform a simulation study to evaluate the robustness of the minimum chi-square method for estimation of the population size, and compare the minimum chi-square method with the full and conditional likelihood

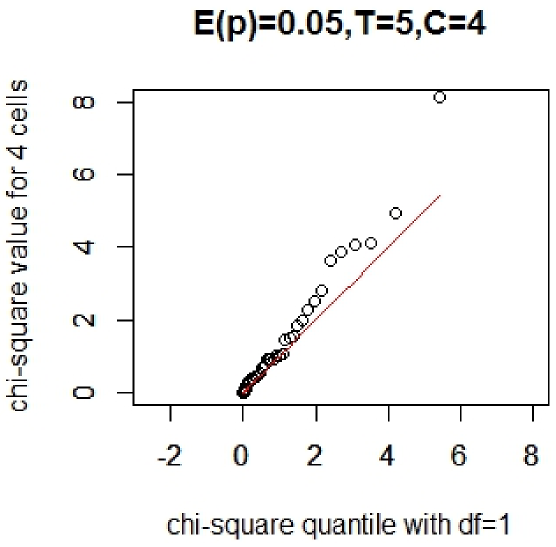

(a)

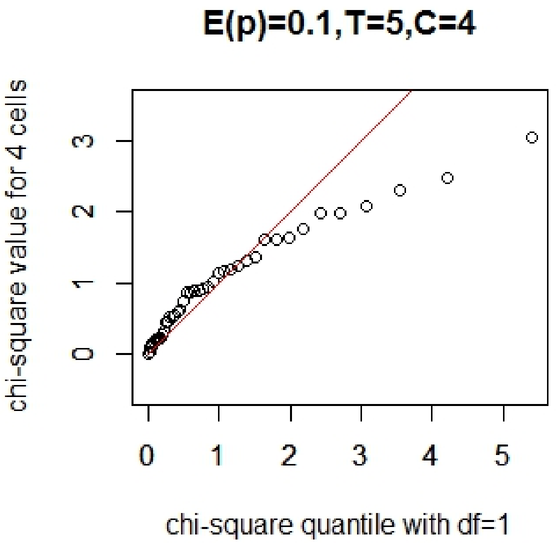

(b)

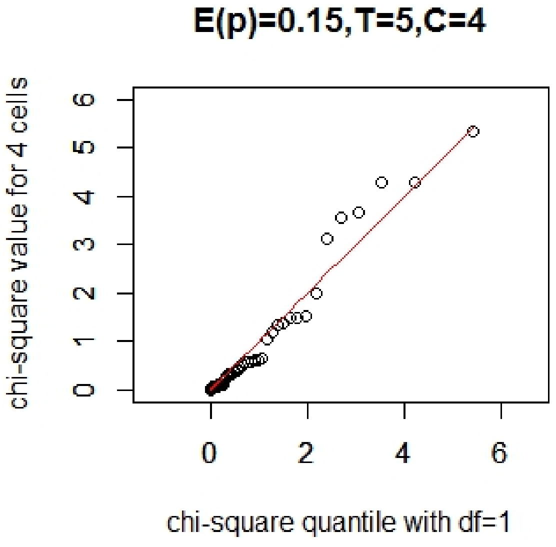

(c)

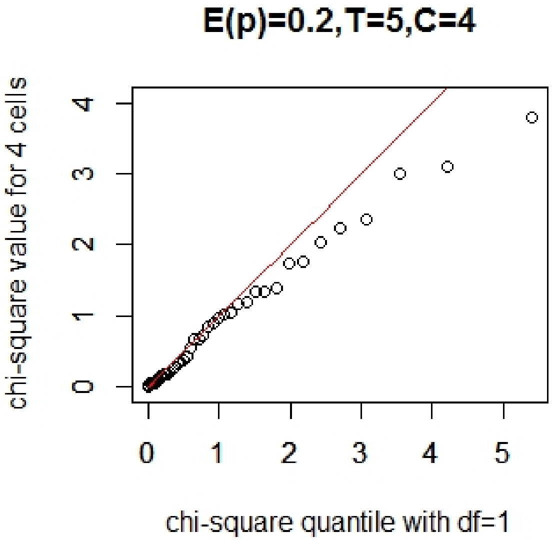

(d)

Fig S1. QQ-plots for $T = 5$, $C = 4$ for (a) $E(p) = 0.05$, (b) $E(p) = 0.10$, (c) $E(p) = 0.15$ and (d) $E(p) = 0.20$ compared with the line $y = x$. Each plot is based on a random sample of $n = 50$ simulated values of the partial minimum χ^2 statistic plotted against the exact χ^2_1 quantiles.

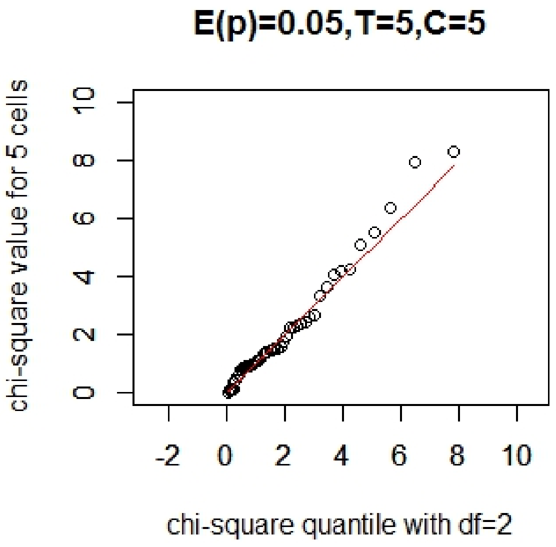

(a)

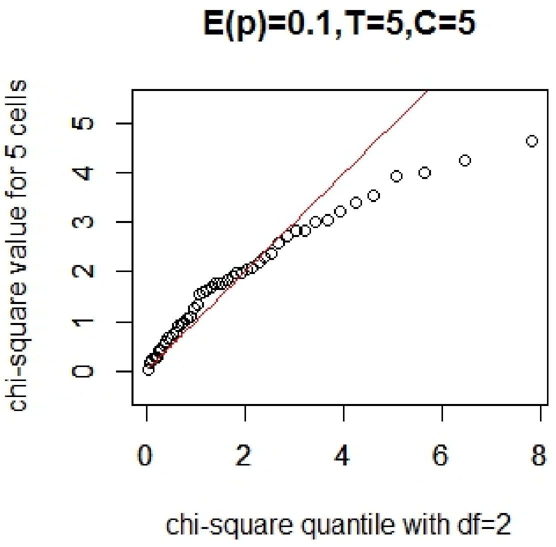

(b)

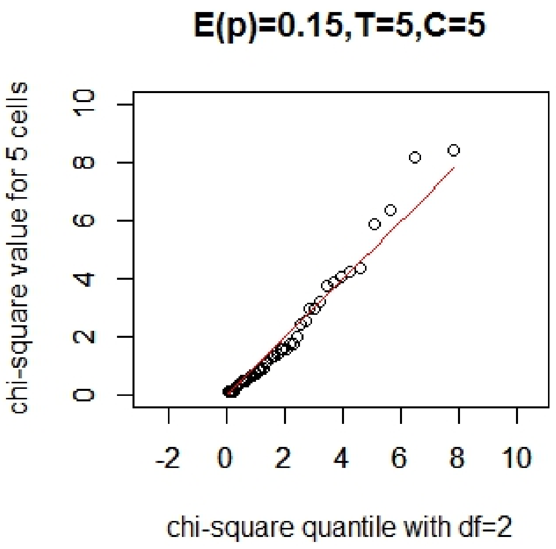

(c)

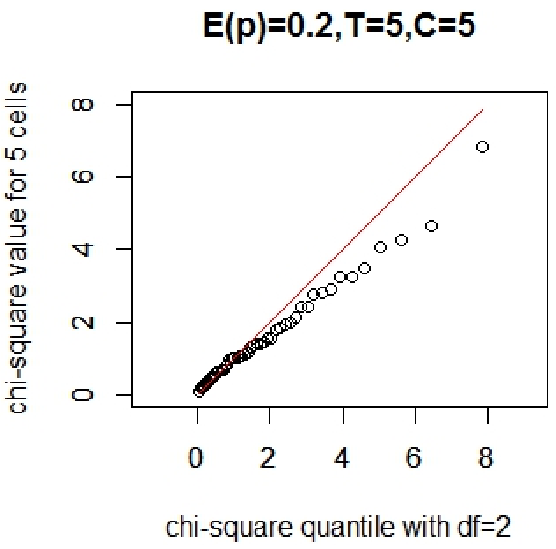

(d)

Fig S2. QQ-plots for $T = 5$, $C = 5$ for (a) $E(p) = 0.05$, (b) $E(p) = 0.10$, (c) $E(p) = 0.15$ and (d) $E(p) = 0.20$ compared with the line $y = x$. Each plot is based on a random sample of $n = 50$ simulated values of the partial minimum χ^2 statistic plotted against the exact χ^2_2 quantiles.

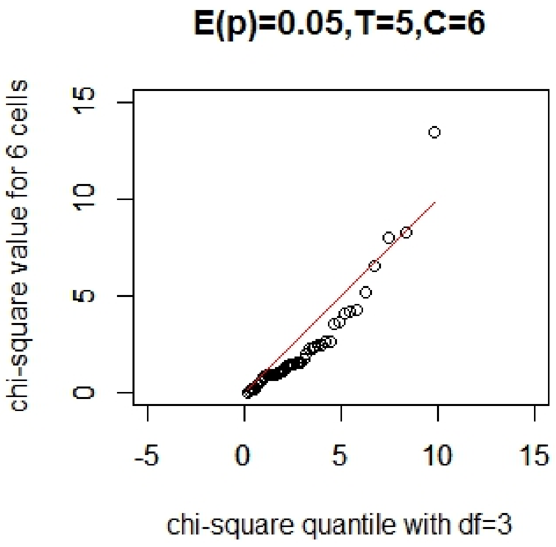

(a)

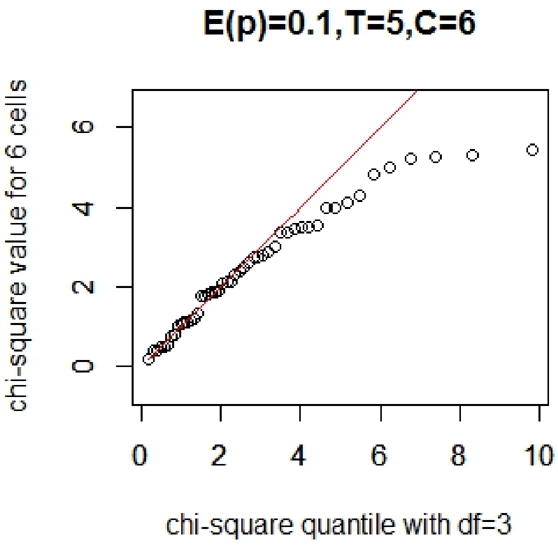

(b)

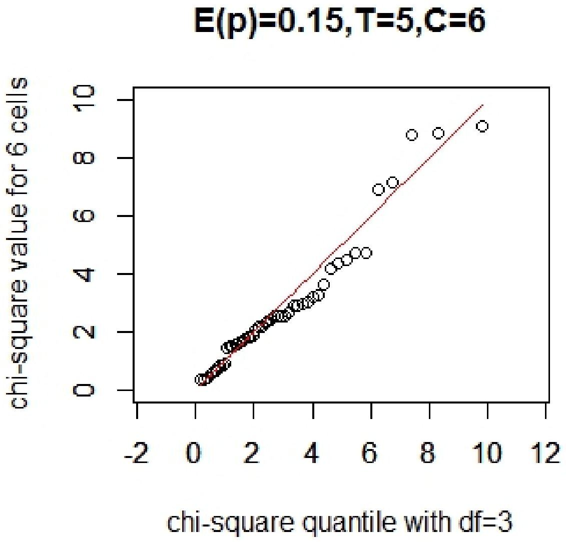

(c)

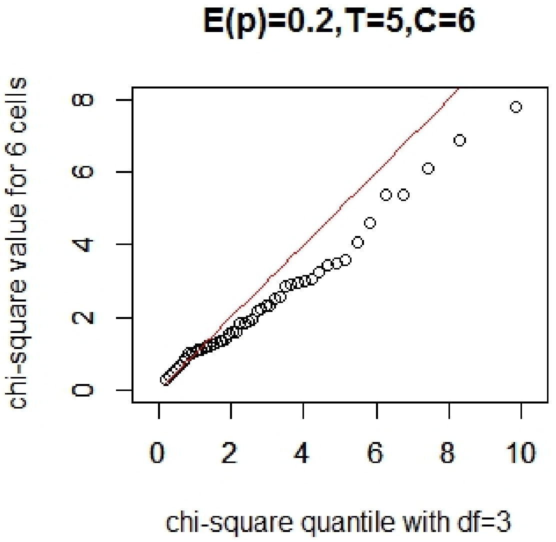

(d)

Fig S3. QQ-plots for $T = 5$, $C = 6$ for (a) $E(p) = 0.05$, (b) $E(p) = 0.10$, (c) $E(p) = 0.15$ and (d) $E(p) = 0.20$ compared with the line $y = x$. Each plot is based on a random sample of $n = 50$ simulated values of the partial minimum χ^2 statistic plotted against the exact χ^2_3 quantiles.

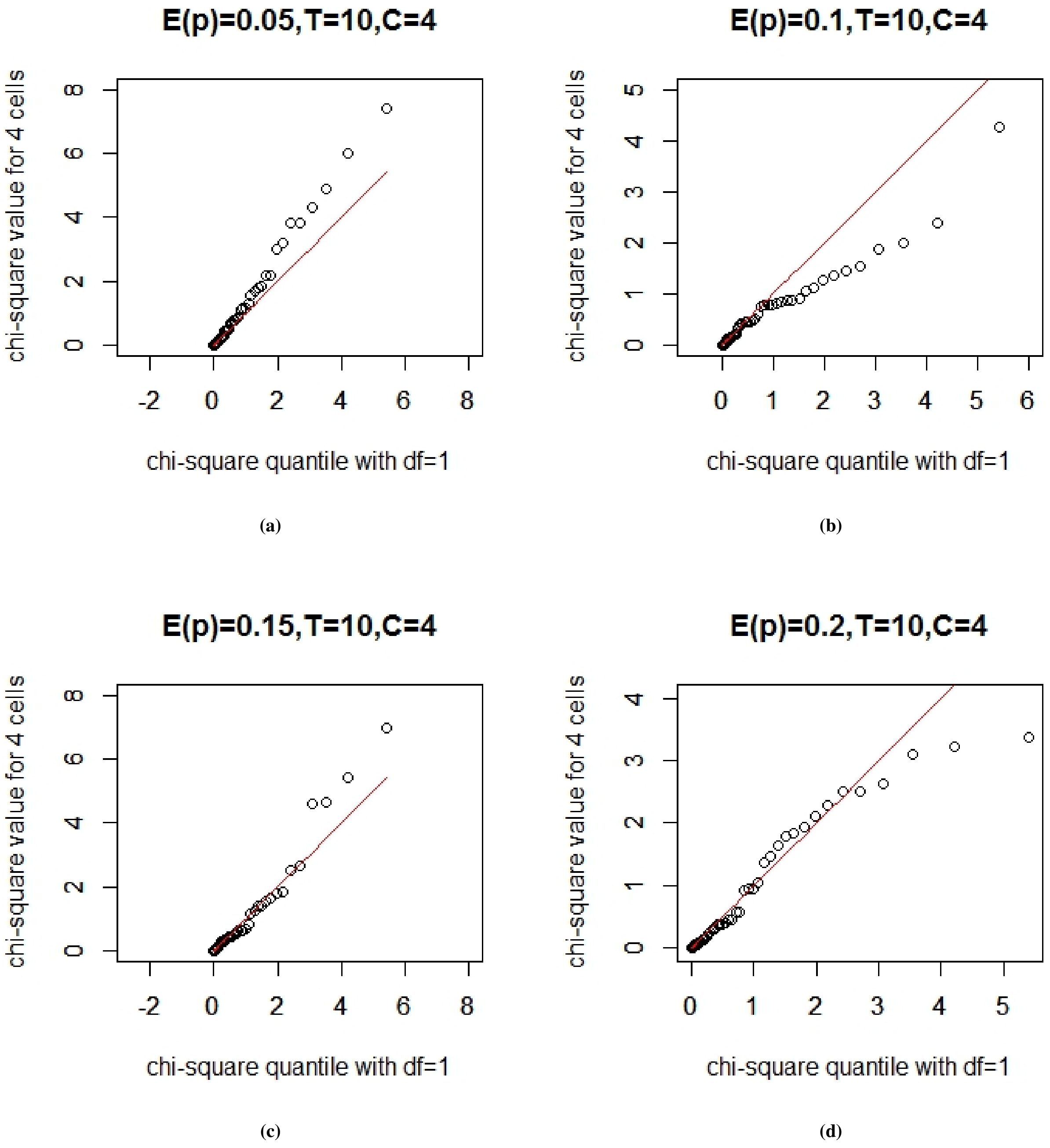

Fig S4. QQ-plots for $T = 10$, $C = 4$ for (a) $E(p) = 0.05$, (b) $E(p) = 0.10$, (c) $E(p) = 0.15$ and (d) $E(p) = 0.20$ compared with the line $y = x$. Each plot is based on a random sample of $n = 50$ simulated values of the partial minimum χ^2 statistic plotted against the exact χ_1^2 quantiles.

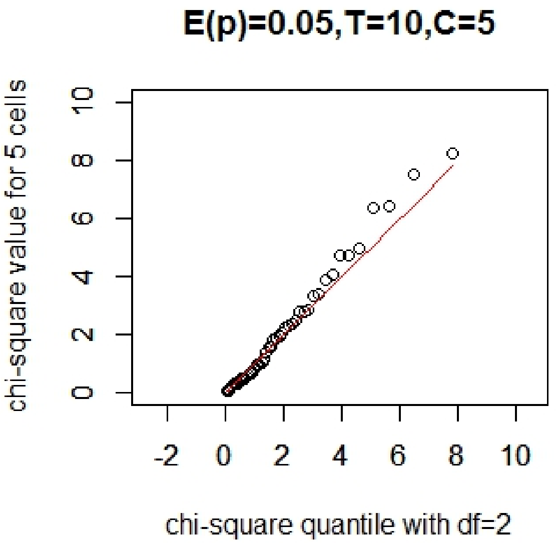

(a)

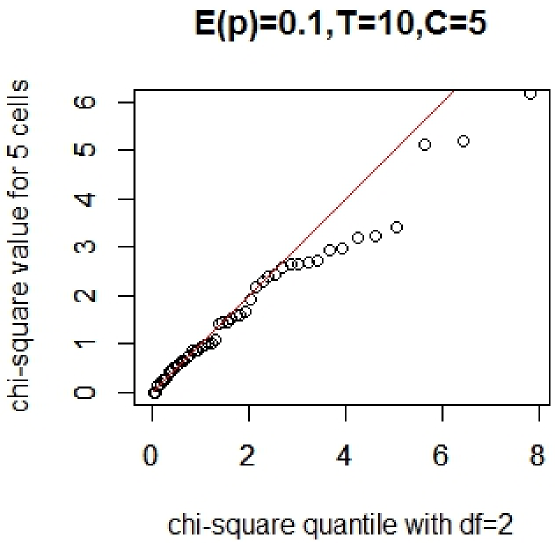

(b)

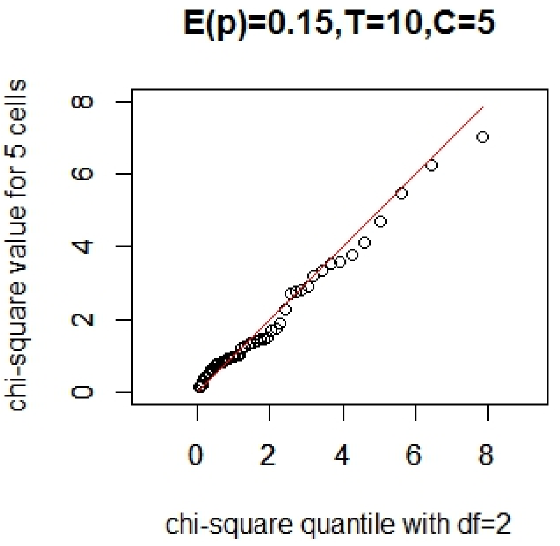

(c)

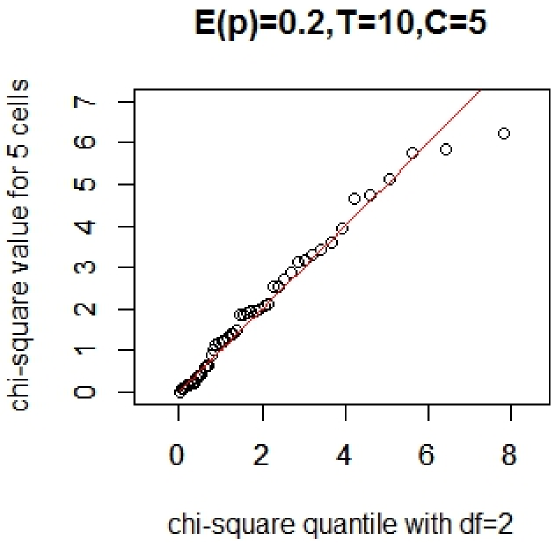

(d)

Fig S5. QQ-plots for $T = 10$, $C = 5$ for (a) $E(p) = 0.05$, (b) $E(p) = 0.10$, (c) $E(p) = 0.15$ and (d) $E(p) = 0.20$ compared with the line $y = x$. Each plot is based on a random sample of $n = 50$ simulated values of the partial minimum χ^2 statistic plotted against the exact χ^2_2 quantiles.

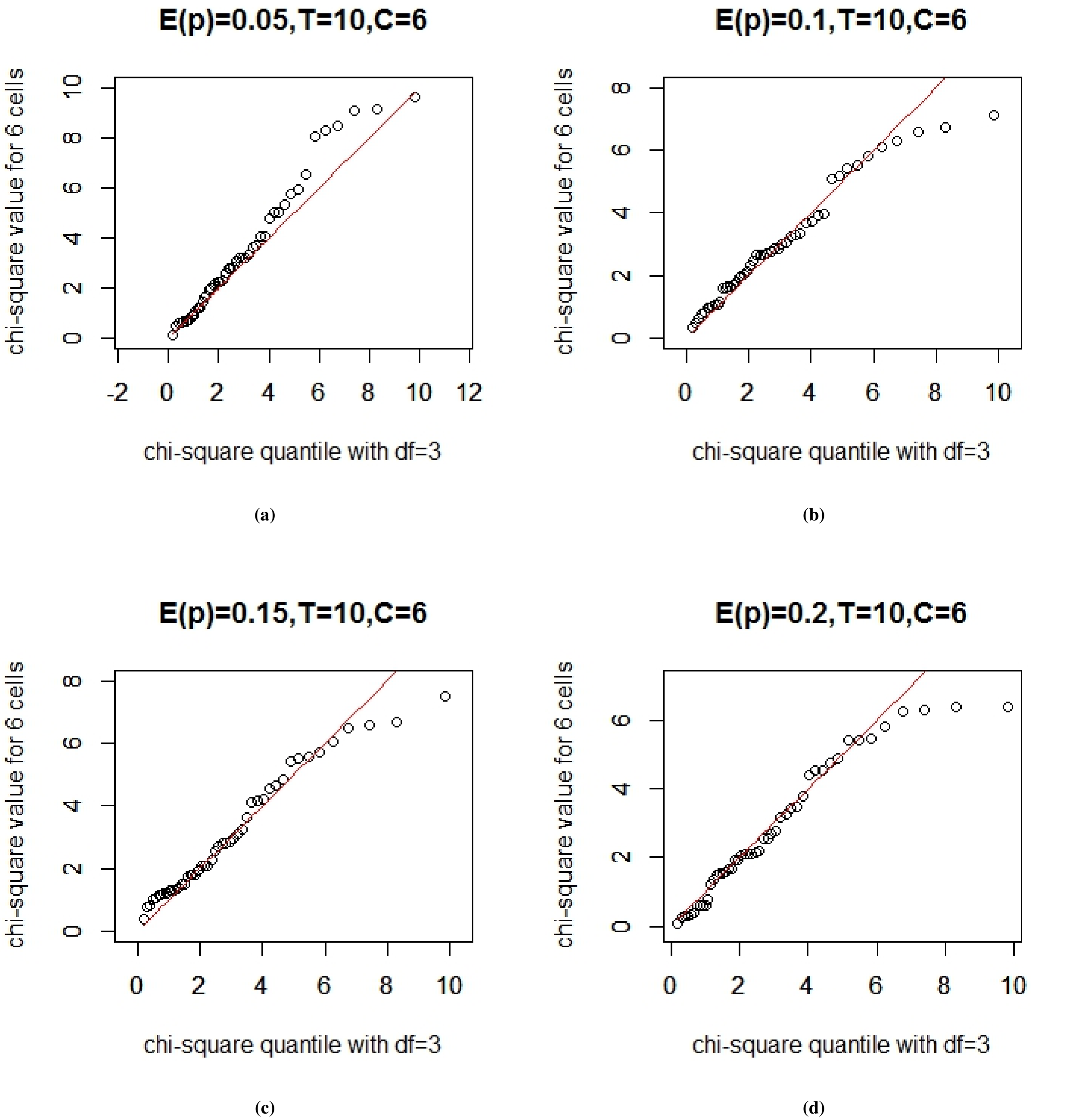

Fig S6. QQ-plots for $T = 10$, $C = 6$ for (a) $E(p) = 0.05$, (b) $E(p) = 0.10$, (c) $E(p) = 0.15$ and (d) $E(p) = 0.20$ compared with the line $y = x$. Each plot is based on a random sample of $n = 50$ simulated values of the partial minimum χ^2 statistic plotted against the exact χ^2_3 quantiles.

methods in terms of robustness. Here, robustness is concerned with the impact of a wrongly specified capture probability model on the accuracy of the estimated population size. Since all methods require a capture probability model in order to estimate the population size, robustness is an issue common to all methods. Thus we investigate which of the three methods is the most robustness in the sense of being the least impacted by the wrong specification of the capture probability model. We do so by comparing the three methods in terms of their bias and MSE under misspecified capture probability models. For simplicity, we set $N = 10000$ and $T = 10$. In each simulation run, we first simulate a set of multistage capture-recapture data under one capture probability model, and then estimate the population size N using the three methods under another (i.e., misspecified) capture probability model. We repeat this process 50 times to obtain 50 estimates for each method. Since we know the true $N = 10000$, we then estimate the bias and MSE of each method by using the 50 estimated values of N, and compare the methods using the estimated bias and MSE.

For convenience, we denote the capture probability model that we use to generate the capture-recapture data by A, and denote the model that we use to estimate the population size by B. A misspecification occurs when $A \neq B$.

2.1 Robustness comparison for the (Beta, Logit-normal) case and the (Beta, 2-point mixture) case

In this subsection, we evaluate the robustness of the three methods against the following misspecifications of the capture probability model (i) $(A, B) =$ (Beta, Logit-normal) and (ii) $(A, B) =$ (Beta, 2-point mixture).

Table S3 provides the numerical results for the case where model A is a Beta(1,9) distribution. The mean of this beta distribution is $E(p) = 0.1$, which represents a population with an average capture probability of 0.1 at each capture occasion. We have also included four different choices of the number of cells for the minimum chi-square method. The 3-cells minimum chi-square method using logit-normal as the capture probability model has the smallest bias and the smallest MSE. The accuracy of the full likelihood and conditional likelihood methods using logit-normal, although they are not as good as that of the 4-cells, 5-cells and 6-cells minimum chi-square methods using logit-normal, are still within the same order of magnitude. The accuracy of all the methods using 2-point mixture as the capture probability model are substantially worse than those based on the logit-normal. Overall, the best minimum chi-square method (the 3-cells method) is more robust than the two likelihood methods in terms of bias and MSE.

Table S3. Robustness comparison for the cases of (A, B) = (Beta(1,9), Logit-normal) and (A, B) = (Beta(1,9), 2-point mixture) with an $E(p) = 0.1$.

Model	Method	Number of Cells	$bias(\hat{N})$	$MSE(\hat{N})$	$\sqrt{MSE(\hat{N})}$
Logit-normal	full likelihood	NA	1329.26	1 823 987.0	1350.55
Logit-normal	conditional likelihood	NA	1335.81	1 826 037.0	1351.31
Logit-normal	chi-square	3	519.36	287 246.3	535.95
Logit-normal	chi-square	4	954.16	1 042 964.0	1021.26
Logit-normal	chi-square	5	1134.81	1 359 969.0	1166.18
Logit-normal	chi-square	6	1248.54	1 606 705.0	1267.56
2-point mixture	full likelihood	NA	1998.56	3 994 343.0	1998.59
2-point mixture	conditional likelihood	NA	2260.25	5 144 931.0	2268.24
2-point mixture	chi-square	3	1607.46	2 815 439.0	1677.93
2-point mixture	chi-square	4	1724.24	3 186 179.0	1784.99
2-point mixture	chi-square	5	1765.59	3 218 138.0	1793.92
2-point mixture	chi-square	6	1904.89	3 653 434.0	1911.40

Table S4 gives the numerical results for the case where model A is a Beta(1.5,8.5) distribution. The mean of this beta distribution is $E(p) = 0.15$, which represents a population with an average capture probability of 0.15 at each capture occasion. With a larger expected capture probability, the simulated capture-recapture data contains more information and this leads to more accurate estimates for all three methods when compared to results in Table S3. The 3-cells minimum chi-square method using logit-normal as the capture probability model again has the smallest bias and the smallest MSE. The accuracy of the full likelihood and conditional likelihood methods using logit-normal, although they are not as good as the 4-cells, 5-cells and 6-cells minimum chi-square methods using logit-normal, are still within the same order of magnitude. The accuracy of methods using 2-point mixture as the capture probability model are again substantially worse than that of methods using the logit-normal.

Table S4. Robustness comparison for the cases of (A, B) = (Beta(1.5,8.5), Logit-normal) and (A, B) = (Beta(1.5,8.5), 2-point mixture) with an $E(p) = 0.15$.

Model	Method	Number of Cells	$bias(\hat{N})$	$MSE(\hat{N})$	$\sqrt{MSE(\hat{N})}$
Logit-normal	full likelihood	NA	652.34	443 919.7	666.27
Logit-normal	conditional likelihood	NA	650.81	441 375.0	664.36
Logit-normal	chi-square	3	55.36	28 854.2	169.87
Logit-normal	chi-square	4	438.50	224 243.7	473.54
Logit-normal	chi-square	5	537.01	312 304.1	558.84
Logit-normal	chi-square	6	587.14	365 428.0	604.51
2-point mixture	full likelihood	NA	1366.71	1 897 777.0	1377.60
2-point mixture	conditional likelihood	NA	1326.84	1 770 334.0	1330.54
2-point mixture	chi-square	3	1125.57	1 658 166.0	1287.70
2-point mixture	chi-square	4	1315.75	1 754 673.0	1324.64
2-point mixture	chi-square	5	936.83	916 295.2	957.23
2-point mixture	chi-square	6	1130.90	1 292 143.0	1136.73

Table S5 gives the numerical results for the case where model A is a Beta(2,8) distribution. The mean of this beta distribution is $E(p) = 0.2$, which represents a population with an average capture probability of 0.2 at each capture occasion. Comparing this table with Tables S3 and S4, we see that observations we made about Table S4 also applies to this table. In particular, the observation that increasing the expected capture probability $E(p)$ (of model A) reduces the bias and MSE of all methods is, at first glance, a bit surprising because model B remains misspecified and so one would expect the increase in $E(p)$ of model A to have little impact on the estimation accuracy. While we do not have a theoretical explanation for this phenomenon, we believe this is because different types of capture probability distributions with high $E(p)$ are in fact quite similar. An extreme example is when $E(p) = 1$, and in this case all models degenerate into the same distribution with a point mass of 1 at 1. Thus, when the $E(p)$ of model A is high, the misspecification problem is less serious because there are members in the B family that are close to such a model A.

Table S5. Robustness comparison for the cases of (A, B) = (Beta(2,8), Logit-normal) and (A, B) = (Beta(2,8), 2-point mixture) with an $E(p) = 0.2$.

Model	Method	Number of Cells	$bias(\hat{N})$	$MSE(\hat{N})$	$\sqrt{MSE(\hat{N})}$
Logit-normal	full likelihood	NA	323.36	113 607.5	337.06
Logit-normal	conditional likelihood	NA	334.80	120 314.7	346.86
Logit-normal	chi-square	3	42.44	12 141.7	110.19
Logit-normal	chi-square	4	178.51	48 179.2	219.50
Logit-normal	chi-square	5	244.33	68 817.8	262.33
Logit-normal	chi-square	6	286.54	89 797.9	299.66
2-point mixture	full likelihood	NA	923.96	857 010.1	925.75
2-point mixture	conditional likelihood	NA	793.38	635 171.3	796.98
2-point mixture	chi-square	3	998.05	1 003 974.0	1001.99
2-point mixture	chi-square	4	777.94	609 968.8	781.01
2-point mixture	chi-square	5	481.76	251 148.0	501.15
2-point mixture	chi-square	6	615.77	389 138.7	623.81

2.2 Robustness comparison for the (Logit-normal, Beta) case

In this subsection, we evaluate the robustness of the three methods when (A, B) = (Logit-normal, Beta). Table S6 shows the numerical results for the case where model A is a Logit-normal($\mu = -2.5, \sigma = 1$) distribution. The mean of this logit-normal distribution is $E(p) \approx 0.1$, which represents a population with an average capture probability of roughly 0.1 at each capture occasion. We have also included four different choices of the number of cells for the minimum chi-square method. The 3-cells minimum chi-square method has the smallest bias and the smallest MSE. The accuracies of the full likelihood and conditional likelihood methods, although they are not as good as that of the 4-cells, 5-cells and 6-cells minimum chi-square methods, are still within the same order of magnitude. Overall, the best minimum chi-square method (the 3-cells method) is more robust than the two likelihood methods in terms of bias and MSE.

Table S6. Robustness comparison for the case of (A, B) = (Logit-normal($\mu = -2.5, \sigma = 1$), Beta) with an $E(p) \approx 0.1$.

Model	Method	Number of Cells	$bias(\hat{N})$	$MSE(\hat{N})$	$\sqrt{MSE(\hat{N})}$
Beta	full likelihood	NA	3082.18	10 210 176.0	3195.34
Beta	conditional likelihood	NA	3325.78	11 847 037.0	3441.95
Beta	chi-square	3	776.23	644 654.4	802.90
Beta	chi-square	4	1746.56	3 975 667.0	1993.91
Beta	chi-square	5	2219.60	5 408 810.0	2325.69
Beta	chi-square	6	2708.35	8 001 593.0	2828.71

Table S7 gives the numerical results for the case where model A is a Logit-normal($\mu = -2, \sigma = 1$) distribution. The mean of this logit-normal distribution is $E(p) \approx 0.15$. Table S8 provides the numerical results for the case where model A is a Logit-normal($\mu = -1.5, \sigma = 1$) distribution. The mean of this logit-normal distribution is $E(p) \approx 0.2$. Again, the 3-cells minimum chi-square is the most robust in terms of having the smallest bias and MSE under misspecification of the capture probability model. In general, the minimum chi-square method, regardless the number of cells used, is more robust than the likelihood based methods. This is expected as the minimum chi-square method does not depend on the correct specification of the capture probability model as much as the likelihood methods.

Table S7. Robustness comparison for the case of (A, B) = (Logit-normal($\mu = -2, \sigma = 1$), Beta) with an $E(p) \approx 0.15$.

Model	Method	Number of Cells	$bias(\hat{N})$	$MSE(\hat{N})$	$\sqrt{MSE(\hat{N})}$
Beta	full likelihood	NA	1530.26	2 493 332.0	1579.03
Beta	conditional likelihood	NA	1538.04	2 492 521.0	1578.77
Beta	chi-square	3	29.96	13 046.8	114.22
Beta	chi-square	4	801.76	806 517.2	898.06
Beta	chi-square	5	1001.03	1 124 906.0	1060.62
Beta	chi-square	6	1205.00	1 570 096.0	1253.04

Table S8. Robustness comparison for the case of (A, B) = (Logit-normal($\mu = -1.5, \sigma = 1$), Beta) with an $E(p) \approx 0.2$.

Model	Method	Number of Cells	$bias(\hat{N})$	$MSE(\hat{N})$	$\sqrt{MSE(\hat{N})}$
Beta	full likelihood	NA	746.38	586 004.8	765.51
Beta	conditional likelihood	NA	754.70	597 386.9	772.91
Beta	chi-square	3	186.76	40 629.8	201.57
Beta	chi-square	4	361.73	191 716.0	437.85
Beta	chi-square	5	493.17	290 046.2	538.56
Beta	chi-square	6	599.78	393 531.5	627.32

2.3 Robustness comparison varying N, $E(p)$, and $g(p; \boldsymbol{\theta})$

In the main article, we were concerned with estimating the size of the white stork population which was found to be around 4000. In the above comparison, we have considered only the large population size of $N = 10,000$. To examine the robustness of the estimation methods when N is smaller, we consider $(A, B) =$ (Beta, Logit-normal) case and $(A, B) =$ (Logit-normal, Beta) when the true population size $N = 1000, 4000, 6000, 8000$ or 10000 and $T = 16$. The parameters of model A are chosen so that the expected capture probability $E(p) = 0.05$ or 0.20.

Tables S9 to S12 contain the bias and MSE of the three methods. We see from these tables that the minimum chi-square methods outperform the likelihood methods in most cases. In particular, when N is large and $E(p)$ is high, the minimum chi-square method outperforms the likelihood methods, regardless the number of cells used. Based on these results, we conclude that the minimum chi-square method is in general more robust than the likelihood methods.

Table S9. Robustness comparison for the case of $(A, B) =$ (Beta(1,19), Logit-normal) with an $E(p) = 0.05$.

N	Method	Number of Cells	$bias(\hat{N})$	Relative bias	$\sqrt{MSE(\hat{N})}$	Relative $\sqrt{MSE(\hat{N})}$
1000	Full	NA	357	36%	597	60%
	Conditional	NA	168	17%	183	18%
	Chi-square	4	60	6%	192	19%
		5	151	15%	171	17%
		6	159	16%	177	18%
		7	161	16%	178	18%
4000	Full	NA	1256	31%	1762	44%
	Conditional	NA	657	16%	675	17%
	Chi-square	4	538	13%	585	15 %
		5	586	15%	612	15%
		6	628	16%	648	16%
		7	637	16%	655	16%
6000	Full	NA	1037	17%	1145	19%
	Conditional	NA	944	16%	964	16%
	Chi-square	4	744	12%	814	14%
		5	830	14%	869	14%
		6	878	15%	905	15%
		7	908	15%	934	16%
8000	Full	NA	1304	16%	1337	17%
	Conditional	NA	1298	16%	1320	16%
	Chi-square	4	953	12%	1044	13%
		5	1102	14%	1141	14%
		6	1182	15%	1210	15%
		7	1240	16%	1263	16%
10000	Full	NA	1558	16%	1580	16%
	Conditional	NA	1590	16%	1611	16%
	Chi-square	4	1284	13%	1340	13%
		5	1416	14%	1455	15%
		6	1548	15%	1573	16%
		7	1603	16%	1623	16%

Table S10. Robustness comparison for the case of $(A, B) =$ (Beta(2,8), Logit-normal) with an $E(p) = 0.2$.

N	Method	Number of Cells	$bias(\hat{N})$	Relative bias	$\sqrt{MSE(\hat{N})}$	Relative $\sqrt{MSE(\hat{N})}$
1000	Full	NA	28	2.8%	32	3%
	Conditional	NA	27	2.7%	31	3%
	Chi-square	4	29	2.9%	35	4%
		5	21	2.1%	27	3%
		6	21	2.1%	26	3%
		7	22	2.2%	26	3%
4000	Full	NA	156	3.9%	161	4%
	Conditional	NA	155	3.9%	160	4%
	Chi-square	4	62	1.5%	76	2%
		5	59	1.5%	72	2%
		6	68	1.7%	79	2%
		7	76	1.9%	85	2%
6000	Full	NA	156	2.6%	161	3%
	Conditional	NA	155	2.6%	160	3%
	Chi-square	4	73	1.2%	92	2%
		5	88	1.5%	102	2%
		6	104	1.7%	115	2%
		7	117	1.9%	125	2%
8000	Full	NA	208	2.6%	212	3%
	Conditional	NA	207	2.6%	211	3%
	Chi-square	4	101	1.3%	118	1%
		5	117	1.5%	127	2%
		6	144	1.8%	151	2%
		7	167	2.1%	173	2%
10000	Full	NA	269	2.7%	274	3%
	Conditional	NA	268	2.7%	273	3%
	Chi-square	4	121	1.2%	140	1%
		5	147	1.5%	165	2%
		6	174	1.7%	184	2%
		7	199	2.0%	208	2%

Table S11. Robustness comparison for the case of (A, B) = (Logit-normal($\mu = -3.3, \sigma = 0.9$), Beta) with an $E(p) \approx 0.05$.

N	Method	Number of Cells	$bias(\hat{N})$	Relative bias	$\sqrt{MSE(\hat{N})}$	Relative $\sqrt{MSE(\hat{N})}$
1000	Full	NA	298	30%	363	36%
	Conditional	NA	514	51%	741	74%
	Chi-square	4	346	35%	655	66%
		5	411	41%	710	71%
		6	452	45%	701	70%
		7	505	51%	750	75%
4000	Full	NA	1779	44%	1950	49%
	Conditional	NA	2033	51%	2417	60%
	Chi-square	4	970	24%	1512	38%
		5	1263	32%	1614	40%
		6	1471	37%	1777	44%
		7	1779	44%	2135	53%
6000	Full	NA	1279	16%	1377	17%
	Conditional	NA	3629	45%	3937	49%
	Chi-square	4	1923	24%	2557	32%
		5	2386	30%	2729	34%
		6	2836	35%	3149	39%
		7	3156	39%	3442	43%
8000	Full	NA	1279	16%	1371	17%
	Conditional	NA	3896	49%	4632	58%
	Chi-square	4	2201	28%	2955	37%
		5	2649	33%	3051	38%
		6	3244	41%	3648	46%
		7	3655	46%	4043	51%
10000	Full	NA	2730	27%	2799	28%
	Conditional	NA	4508	45%	4766	48%
	Chi-square	4	2096	21%	2542	25%
		5	2967	30%	3272	33%
		6	3535	35%	3798	38%
		7	3935	39%	4176	42%

Table S12. Robustness comparison for the case of (A, B) = (Logit-normal($\mu = -1.5, \sigma = 1$), Beta) with an $E(p) \approx 0.2$.

N	Method	Number of Cells	$bias(\hat{N})$	Relative bias	$\sqrt{MSE(\hat{N})}$	Relative $\sqrt{MSE(\hat{N})}$
1000	Full	NA	30	3%	95	9%
	Conditional	NA	52	5%	61	6%
	Chi-square	4	20	2%	55	5%
		5	21	2%	40	4%
		6	28	3%	43	4%
		7	34	3%	47	5%
4000	Full	NA	130	3%	178	4%
	Conditional	NA	225	6%	231	6%
	Chi-square	4	81	2%	122	3%
		5	106	3%	129	3%
		6	133	3%	146	4%
		7	153	4%	163	4%
6000	Full	NA	219	4%	247	4%
	Conditional	NA	333	6%	339	6%
	Chi-square	4	109	2%	153	3%
		5	144	2%	166	3%
		6	186	3%	199	3%
		7	220	4%	230	4%
8000	Full	NA	337	4%	373	5%
	Conditional	NA	439	5%	447	6%
	Chi-square	4	145	2%	194	2%
		5	193	2%	213	3%
		6	243	3%	256	3%
		7	291	4%	302	4%
10000	Full	NA	575	6%	593	6%
	Conditional	NA	555	6%	562	6%
	Chi-square	4	185	2%	242	2%
		5	243	2%	268	3%
		6	310	3%	325	3%
		7	365	4%	376	4%
